# The Glutaminase-Dependent Acid Resistance System: Qualitative and Quantitative Assays and Analysis of Its Distribution in Enteric Bacteria

**DOI:** 10.3389/fmicb.2018.02869

**Published:** 2018-11-15

**Authors:** Eugenia Pennacchietti, Chiara D'Alonzo, Luca Freddi, Alessandra Occhialini, Daniela De Biase

**Affiliations:** ^1^Department of Medico-Surgical Sciences and Biotechnologies, Laboratory Affiliated to the Istituto Pasteur Italia-Fondazione Cenci Bolognetti, Sapienza University of Rome, Latina, Italy; ^2^Institut de Recherche en Infectiologie de Montpellier, CNRS, University of Montpellier, Montpellier, France

**Keywords:** acid stress, *Escherichia coli*, GadC antiporter, glutamine, GABA, rapid assay, HPLC

## Abstract

Neutralophilic bacteria have developed several strategies to overcome the deleterious effects of acid stress. In particular, the amino acid-dependent systems are widespread, with their activities overlapping, covering a rather large pH range, from 6 to <2. Recent reports showed that an acid resistance (AR) system relying on the amino acid glutamine (AR2_Q), the most readily available amino acid in the free form, is operative in *Escherichia coli, Lactobacillus reuteri*, and some *Brucella* species. This system requires a glutaminase active at acidic pH and the antiporter GadC to import *L*-glutamine and export either glutamate (the glutamine deamination product) or GABA. The latter occurs when the deamination of glutamine to glutamate, via acid-glutaminase (YbaS/GlsA), is coupled to the decarboxylation of glutamate to GABA, via glutamate decarboxylase (GadB), a structural component of the glutamate-dependent AR (AR2) system, together with GadC. Taking into account that AR2_Q could be widespread in bacteria and that until now assays based on ammonium ion detection were typically employed, this work was undertaken with the aim to develop assays that allow a straightforward identification of the acid-glutaminase activity in permeabilized bacterial cells (qualitative assay) as well as a sensitive method (quantitative assay) to monitor in the pH range 2.5–4.0 the transport of the relevant amino acids *in vivo*. The qualitative assay is colorimetric, rapid and reliable and provides several additional information, such as co-occurrence of AR2 and AR2_Q in the same bacterial species and assessment of the growth conditions that support maximal expression of glutaminase at acidic pH. The quantitative assay is HPLC-based and allows to concomitantly measure the uptake of glutamine and the export of glutamate and/or GABA via GadC *in vivo* and depending on the external pH. Finally, an extensive bioinformatic genome analysis shows that the gene encoding the glutaminase involved in AR2_Q is often nearby or in operon arrangement with the genes coding for GadC and GadB. Overall, our results indicate that AR2_Q is likely to be of prominent importance in the AR of enteric bacteria and that it modulates the enzymatic as well as antiport activities depending on the imposed acidic stress.

## Introduction

By definition, neutralophilic microorganisms are those that best grow in the pH range 5–8 (Krulwich et al., 2011). However, during their lifecycle they can be exposed, transiently or for prolonged periods, to acidic conditions (Lund et al., 2014). An increase in extracellular proton concentration such as that found in some areas of the animal host, i.e., the pH ≤ 2.5 in the stomach and, intracellularly, the pH 4.5–5.5 in the phagolysosome, typically challenges the microorganisms, regardless of whether they are pathogens or not. Moreover, many organic acids are used as food preservative (Hazan et al., 2004; Theron and Lues, 2010) and their production is a common strategy adopted by food-fermenting bacteria to exert a control of other microbial species in food matrices (Ross et al., 2002; Krulwich et al., 2011) and/or in the distal gut (Flint et al., 2008). Thus, perceiving and responding to an acidic environment is important in microbial physiology because it ensures survival in a microbial community and in the host (Krulwich et al., 2011; Lund et al., 2014).

The acid-protecting systems discovered to date allow the microorganisms to counteract the deleterious effect resulting from an intracellular increase of protons which negatively affect the transmembrane potential, by dissipating it, the activity of many enzymes, and the folding of proteins. In other words, acids can be a threat to microbial vitality. On the other hand, it should also be recalled that the acidic pH is a cue that triggers virulence trait in pathogenic microorganisms (Porte et al., 1999; Vandal et al., 2009; De Biase and Lund, 2015).

In Gram-positive and Gram-negative neutralophilic bacteria protection from acidic stress is provided by many systems each acting in a precise pH-range and having specific requirements to function. The pH range of activity of these systems is such that they effectively protect from the life-threatening effects of the exposure to acid stress in a rather large pH range, from pH 6 to pH 2.5 or lower (Kanjee and Houry, 2013; Lund et al., 2014). Among the strategies adopted to face the acid encounter, the amino acid-dependent systems have been shown to be quite widespread in enteric bacteria exposed to harsh acid. In particular, the glutamate-dependent acid resistance (GDAR; also named AR2) system is extremely powerful: it provides the most effective protection to the bacterial cells exposed to a pH ≤ 2.5, up to some hours (Lin et al., 1996).

**Figure 1:**
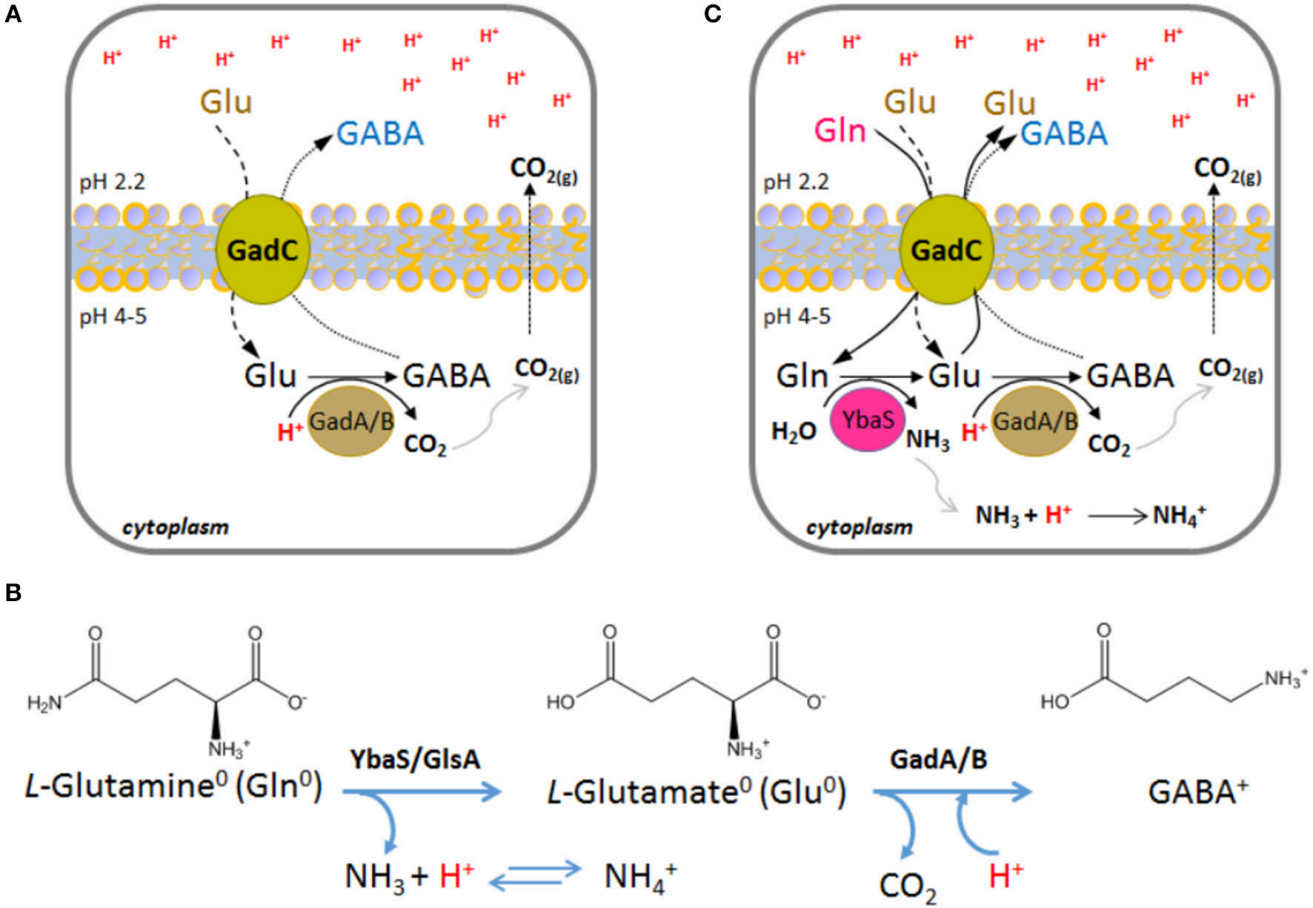
Schematic representation of the enzymatic reactions and transport activity in the AR2 and AR2_Q systems in *Escherichia coli*. **(A)** A typical situation where the AR2 comes into action is when the drop of the extracellular pH to below 2.5 leads, as a consequence, to the decrease of the intracellular pH to below 5. This activates the GadC-mediated import of Glu^0^ (if available extracellularly) and the export of GABA^+^, the latter generated by the intracellular proton-consuming activity of GadA/B. **(B)** Chemical structures and net charge of the species imported and exported by GadC, according to (Ma et al., 2013; Tsai et al., 2013). The reactions and the enzymes involved are shown. **(C)** A situation where the AR2_Q can be operative is when the extracellular pH drops below 2.5 and, as a consequence, the intracellular pH decreases to below 5. This activates the GadC-mediated import of Gln^0^ (when available extracellularly) and the export of Glu^0^ (arising from Gln deamination) or GABA^+^ (arising from Glu decarboxylation). In AR2_Q, protons are taken up during ammonium ion formation and during GABA synthesis. According to previous reports (Ma et al., 2013; Tsai et al., 2013), GadC can export either Glu^0^ or GABA^+^, depending on the cellular need to buffer the cytosol and effectively consume protons.

This system is operative in *Escherichia coli*, as well as in many other bacteria (De Biase and Pennacchietti, 2012; Occhialini et al., 2012; Damiano et al., 2015). It relies on the activity of the pyridoxal 5′-phosphate (PLP)-dependent enzyme glutamate decarboxylase (two isoforms, GadA and GadB, are present in *E. coli*, whereas in other microorganisms only one glutamate decarboxylase, GadB, occurs) and on the activity of the plasma membrane antiporter GadC, which imports glutamate (Glu) and export its decarboxylation product, i.e., γ-aminobutyrate (GABA; Figure 1A). Both, the decarboxylase and the antiporter are maximally active in the pH range 4.0–5.5 and are inactive at pH ≥ 6.5 (De Biase et al., 1996; Capitani et al., 2003; Gut et al., 2006; Ma et al., 2012; Grassini et al., 2015). Strikingly, in both proteins, i.e., GadB and GadC, the C-terminal tail of each polypeptide chain is responsible for self-inhibition at pH ≥ 6.5 (Gut et al., 2006; Pennacchietti et al., 2009; Ma et al., 2012, 2013). Moreover, while the only known physiological substrate of *E. coli* GadA/B is Glu (Fonda, 1972), GadC is able to import glutamine (Gln) in addition to Glu (Ma et al., 2012, 2013; Tsai et al., 2013). It was elegantly shown that GadC provides a “selective transport path” for Gln, Glu and GABA, according to their overall net charge, and this is instrumental to avoid further proton stress to the cell. Specifically, GadC allows the influx of Glu^0^ and Gln^0^ and the efflux of Glu^0^ or GABA^+^ (Ma et al., 2013; Tsai et al., 2013) (Figure 1B). In particular, the Glu^0^(or Gln^0^)_in_/GABA${}_{\text{out}}^{+}$ pH-dependent activity of GadC is responsible for pumping out positive charges, is positively affected by a positive membrane potential, and decreases when the membrane potential is negative or nil (Ma et al., 2013; Tsai et al., 2013).

The ability of GadC to import Gln, in exchange for Glu or GABA, provided the first evidence that glutamine can be a physiological substrate for GadC (Ma et al., 2012). This finding was reinforced by the same authors who demonstrated that *E. coli* possesses a glutamine-dependent AR system relying on the availability of extracellular glutamine, typically in the free form in many food sources, and on the enzyme YbaS, a glutaminase active in the acidic pH range (Lu et al., 2013) (Figure 1C). The glutamine-dependent AR system was therefore renamed AR2_Q, as it shares GadC with the AR2 system (Lund et al., 2014).

Recent reports have demonstrated the occurrence of an AR2_Q in other bacteria than *E. coli* (Lu et al., 2013), such as *Lactobacillus reuteri* (Teixeira et al., 2014) and some *Brucella* species (Freddi et al., 2017). In all these species, the AR2_Q provides protection to extreme acid stress, i.e., pH 2.5, to the same extent as the powerful system depending on Glu (AR2), provided that glutamine was available extracellularly.

Until now the activity of the acid glutaminase, YbaS, was measured by detecting ammonia production either directly, through an ammonium ion electrode (Lu et al., 2013), or indirectly, using a coupled assay with glutamate dehydrogenase (Brown et al., 2008). This work was therefore undertaken with the aim to develop assays that allow, on one hand, a rapid and reliable identification of acid-glutaminase activity in permeabilized bacterial cells (qualitative assay) and, on the other hand, a sensitive HPLC-based method for the detection of extracellular Gln, Glu, and GABA levels *in vivo* (quantitative assay). The latter assay allowed also to show that, in the pH range 2.5–4.0, *E. coli* modulates the enzymatic as well as the antiport activities depending on the imposed acidic stress. Moreover, by means of an extensive bioinformatic analysis it was possible to show that the gene coding for the acid-glutaminase is often found nearby, likely in an operon arrangement, with the genes coding for GadC and GadB, thus suggesting that AR2_Q is as widespread in bacteria, mostly Gram-negative and enteric, as AR2.

## Materials and methods

### Materials

Restriction enzymes, alkaline phosphatase, and ampicillin were obtained from Roche. The DNA Blunt T/A ligase and Taq-Q5 High-Fidelity DNA polymerase were from New England Biolabs. Plasmid DNA preparation and DNA extraction from agarose gel were carried out using PureYield™ Plasmid miniprep system (Promega) and QIAEX II Gel Extraction Kit (Qiagen), respectively.

Ingredients for bacterial growth were from Difco. Bromocresol green was from Fluka; L-Glutamic acid, L-Glutamine, D,L-Norvaline, sodium acetate, sodium chloride, Triton X-100, GABA, and other reagents, if not otherwise stated, were from Sigma–Aldrich. Super gradient grade methanol for HPLC was from VWR. 2-*o-*phthalaldehyde (OPA) was from Agilent Technologies. Oligonucleotide synthesis and DNA sequencing services were by MWG Biotech.

### Bacterial strains, plasmids and growth conditions

The bacterial strains, plasmids, and oligonucleotides used in this work are listed in Table S1. *E. coli* K12 strain MG1655 and its isogenic mutant derivatives Δ*ybaS*, Δ*gadC*, and Δ*gadAB* as well as the complemented strains were reported elsewhere or prepared as part of this work (Occhialini et al., 2012; Freddi et al., 2017; Table S1).

Depending on the experiments to be carried out, the *E. coli* strains were grown in the following media at 37°C: LB broth (Luria Bertani), pH 7.4; LBG, pH 5.0 (LB acidified to pH 5.0 with HCl and supplemented with 0.4% glucose); LB-MOPS, pH 8.0 (LB buffered at pH 8.0 with 100 mM morpholinepropanesulfonic acid, MOPS); LBG-MOPS, pH 8.0 (as LB-MOPS, pH 8.0, and supplemented with 0.4% glucose). All the *Brucella* strains were grown on TS (Tryptic Soy, Difco) agar and broth at 37°C in a biosafety level laboratory L3 in the respect of the legislation (BSL3 confinement; IRIM Montpellier). *Yersinia ruckeri* was grown in NB (Nutrient Broth, Difco) at 25°C. When required, the antibiotics ampicillin, kanamycin, and chloramphenicol were added at the concentration of 100, 25, and 34 μg/ml, respectively.

### Construction and complementation of *E. coli* Δ*ybas* mutant strains

The Δ*ybaS* mutant strain was obtained from *E. coli* using the one-step gene inactivation procedure described by Datsenko and Wanner (2000), plasmid pKD13 as template for amplification of the kanamycin cassette (Kan^R^) and the *ybaS*-specific oligonucleotide pair Δ*ybaS*_for(P1)/Δ*ybaS*_rev(P4) listed in Table S1. The insertion of the Kan^R^ cassette, replacing the *ybaS* gene, was checked by PCR using the primer pair k1/*ybaS*_rev (Table S1).

To complement *E. coli* Δ*ybaS*, plasmids pBBR-*ybaS_Ec*(long), pBBR-*ybaS_Ec*, and pBBR-*glsA_Bm* were used. The construction of these plasmids is described in section Cloning of the *E. coli ybaS* gene in plasmid pBBR1MCS and in Table S1.

### Cloning of the *E. coli ybaS* gene in plasmid pBBR1MCS

In order to assess the influence of the *yba*S promoter on the expression of this gene as directed by plasmid pBBR1MCS, the construct pBBR-*ybaS*_*Ec*, derived from the previously described pBBR-*ybaS*_*Ec*(long) (Freddi et al., 2017 and Table S1), was generated. Briefly, the 110 bp promoter region of *E. coli ybaS* was removed by digesting pBBR-*ybaS*_*Ec*(long) with the restriction enzymes *Xho*I*-Bam*HI, cutting at 159 and 49 bp from the *ybaS* ATG start codon, respectively (Petersen and Moller, 2000). The restriction site for *Bam*HI was introduced as part of the synthetic gene preparation of *ybaS*_*Ec*(long) (Freddi et al., 2017) by replacing the CA in sequence GGCACC with an AT, thereby yielding the sequence GGATCC recognized by *Bam*HI. After restriction with *Xho*I*-Bam*HI, the plasmid was filled-in using Taq-Q5 High-Fidelity DNA polymerase (New England Biolabs) and religated with Blunt T/A ligase (New England Biolabs). The newly generated plasmid, named pBBR-*ybaS*_*Ec*, was sequenced on both strands (MWG Biotech).

### Rapid glutaminase assay (“GlsAssay”)

The “GlsAssay” solution consists of 1 g/L *L*-Glutamine, 0.05 g/L bromocresol green, 90 g/L NaCl, and 3 ml/L Triton X-100 dissolved in distilled water brought to pH 3.1 with KOH. At this pH the starting solution is yellow. The reagent is stable for up to 3 month when stored at 4°C.

It is possible to perform the “GlsAssay” in two ways: (a) from bacterial colonies taken directly from an agar plate or (b) from bacterial cultures grown overnight in liquid media.

#### GlsAssay from colonies on plate

One centimeter (in length) of confluent bacterial colonies or 5 independent colonies (1–4 × 10^8^ cells) were resuspended in 500 μl physiological saline solution (0.9% NaCl in water), to achieve a suspension of 0.2–1.0 × 10^9^ cells/ml. The suspension was centrifuged at 14,000 rpm for 5 min, the supernatant was discarded and the pellet resuspended in 150 μl of the “GlsAssay” solution by vigorous vortexing or pipetting. After incubation for 2 h at 37°C, the change in color from yellow (negative, -) to green (positive, +) or blue (strongly positive, ++) was assessed by eye (see Figure S2).

#### GlsAssay from liquid culture

To set up this assay, bacterial cells were grown at 37°C overnight in 1–2 ml of different media at different pH (i.e., LB, pH 7.4; LBG, pH 5.0; LB-MOPS, pH 8.0; LBG-MOPS, pH 8.0). After 24 h, the cells were harvested by centrifugation at 4,000 rpm for 20 min, resuspended in an isovolume of physiological saline solution and again centrifuged. The latter two steps were repeated one more time. The cell density was then measured and adjusted to an OD_600_ = 2.0 by adding saline solution, where required. 500 μl of each sample at OD_600_ = 2.0, (which corresponds to 0.2–1.0 × 10^9^ cells/ml) were centrifuged at 14,000 rpm for 5 min and the pellet resuspended by vigorous vortexing or pipetting in 150 μl of “GlsAssay” solution. Following the incubation at 37°C for 30 min, 2 h or overnight, the change in color was assessed by eye, as described above.

### Acid resistance (AR) assay and cell viability

#### Acid resistance assay

Depending on the experiment to be performed and for comparison with previous studies (De Biase et al., 1999; Lu et al., 2013), bacteria were grown to the stationary phase for a total of 24 h at 37°C in different media (LB, pH 7.4; LBG, pH 5.0; LB-MOPS, pH 8.0; LBG-MOPS, pH 8.0). The following day the cultures were diluted 1:1000 (except for cultures grown in LBG-MOPS, which were diluted 1:4000) in pre-warmed EG medium, pH 2.2, with/without glutamine (1–3 mM). The acid challenge was carried out for 2 h (*t* = 2 h) at 37°C (without shaking). At this time point, 20 μl of each samples were diluted in physiological saline solution (1:50) and bacteria (20 μl) were plated out and incubated overnight (15–18 h) at 37°C. Only for bacteria challenged in EG without glutamine, plating at *t* = 2 h was without dilution. The percentage of survival was calculated comparing the bacterial counts at *t* = 2 h with the bacterial count at time 0 (*t* = 0). At least three independent experiments (in duplicate) were performed for each strain/condition.

#### Cell viability assay

For the purpose of the HPLC assay described in section HPLC Analysis of Glutamine, Glutamate, and GABA in spent media, cell viability was carried out. It consisted in checking the bacterial counts following incubation for 1 h at pHs less extreme than that used for AR assay, i.e., pH 2.5, pH 3.1, pH 3.5, and pH 4.0, and always in the presence of 3 mM Gln.

### HPLC analysis of glutamine, glutamate and GABA in spent media

In order to assess the contribution of *ybaS*/*glsA, gadAB*, and *gadC* genes to glutamine metabolism and transport in *E. coli* in acidic media, a HPLC-based method to detect simultaneously the three amino acids in the spent media was developed.

#### Sample preparation

Few colonies of each bacterial strain, from freshly streaked LB Agar plates, were inoculated in 2 ml of LBG-MOPS pH 8.0 and grown for 24 h at 37°C. Following growth, each culture was harvested by centrifugation, washed twice with an isovolume (2 ml) of physiological saline solution and finally adjusted to OD_600_ = 4.0 (2.0 × 10^9^ cells/ml). 400-μl aliquots of each culture (now at an identical OD_600_) were then centrifuged and resuspended in EG medium (adjusted with 6N HCl to the indicated pH) containing 3 mM glutamine. At time 0 and after 1 h of incubation at 37°C, the cells were spun down by centrifugation at 4°C for 3–5 min at 10,000 rpm and 100 μl-aliquots of the supernatant (spent medium) were transferred into a clean tube and stored at 4°C for subsequent HPLC analysis, which was always within 3 days from sample collection. A 3.5-μl aliquot from each sample was then added of 10 μL of 0.2 mM Norvaline (internal standard) and HPLC-grade water to reach a final volume of 100 μl.

#### HPLC conditions

HPLC analysis was performed on an Agilent 1260 series system (Agilent Technologies, USA) equipped with a quaternary pump and a fluorescence detector. GABA, Gln, Glu and the internal standard, Norvaline, were separated under a mix of isocratic and gradient elution using an Agilent Poroshell 120 HPH-C18, 4.6 × 150 mm, 2.7 μm LC column (Agilent Technologies) and an Agilent InfinityLab Poroshell UHPLC-C18 guard column (4.6 × 5 mm, 2.7 μm particle size). The mobile phase consisted of Solvent A (50 mM Sodium Acetate buffer: Methanol; 50:50, v/v) adjusted to pH 4.18 with HCl and Solvent B (100% Methanol). Prior to methanol addition, all solutions were filtered through a 0.2-μm nitrocellulose Whatman filters (Ø 47 mm).

Pre-column derivatization of standard amino acids and samples with *o*-phtalaldehyde (OPA) reagent solution (Agilent, 10 mg/ml) was set to be carried out automatically in the HPLC autosampler. Briefly, the derivatization was performed with a programmable automatic injector (Agilent) by mixing 80 μL of sample (or standard solution) with 8 μl of OPA. After 1 min at RT, a 80-μl aliquot of the derivatized sample was directly injected into the HPLC column equilibrated with Solvent A. The elution was carried out at RT and a flow rate of 1 ml/min with the following programme: from 0 to 18 min in 100% Solvent A (isocratic step), from 18.1 to 25 min gradient step to attain 100% Solvent B. The column was then washed for 7 min at 100% Solvent B and equilibrated back into 100% Solvent A with a 4-min gradient step and kept in the same solvent for 10 min, ready for the next injection. The fluorescence detector was set at an excitation wavelength of 240 nm and an emission wavelength of 450 nm according to Perucho et al. (2015).

Using the above HPLC programme, Gln, Glu, GABA, and Norvaline displayed the following retention times: Gln, 2.75 ± 0.02 min; Glu, 4.46 ± 0.06 min; GABA, 12.15 ± 0.20 min; Norvaline, 23.42 ± 0.05 min. The software used for analysis and peak integration was OpenLab (Agilent). Sample peak areas were typically measured by the automatic integration system, though in some circumstances a manual correction of the baseline was required. In order to quantify the amino acids concentrations, the peak area was compared to a calibration curve standard.

#### Preparation of stock and working solutions

300 mM stock solutions of either GABA or Gln or Glu or norvaline were prepared in HPLC-grade water, aliquoted out and stored at 4°C. Working solutions (10 μM) were prepared weekly (every 3 days for norvaline) by diluting the stock solutions either in HPLC-grade water or in EG medium pH 2.5, aliquoted out and stored at 4°C until use.

### Bioinformatics

In order to verify the presence of the *ybaS*/*glsA* gene in the genomes of bacterial species potentially capable of employing GadC to import glutamine for AR2_Q, i.e., possessing *gadBC* operon (alike *E. coli, B. microti*, and *L. reuteri*), a Protein Cluster search (https://www.ncbi.nlm.nih.gov/proteinclusters/) using “glutaminase” as search word we performed.

Eighty Protein Clusters were retrieved, 35 of which were excluded because unrelated to the acid-glutaminase, i.e., because they referred to the “pyridoxal 5'-phosphate synthase glutaminase subunit PdxT.” In addition to this, all the Protein clusters that included proteins shorter than 200 amino acids or longer than 400 amino acids were also excluded because these proteins share little sequence identity with the reference proteins (*E. coli* YbaS, *B. microti* GlsA) and much different in size. This led to restrict the analysis to 20 protein clusters for a total of 1,584 genomes. When possible, redundant genomes were removed.

In checking all the genomes, the above selection criterion (i.e. occurrence of the *gadBC* operon) was made less restrictive by taking into account also those organisms (and genomes) that did not possess GadB (therefore lack the AR2 system) though still possessed a GadC antiporter, making the AR2_Q system still likely to occur. However, in the latter case only those organisms in which the *gadC* and *ybaS/glsA* genes were in close proximity were considered.

All the genomes of interest were therefore searched using the Bacterial Bioinformatics Resource Center PATRIC (https://www.patricbrc.org/) in order to obtain information on the localization of the relevant genes in the genome, their orientation and relative distances, also in a visual way. For completeness of information, when available, fully sequenced genomes were selected among a given species.

### Statistical analysis

Data from AR assays were analyzed via “two-way ANOVA” using the Bonferroni test (as available in the GraphPad Prism software suite, version v5.0a). Data were expressed as means of three independent experiments with standard deviations. Differences were considered statistically significant when *P* < 0.05.

## Results

### The “GlsAssay” provides a rapid and reliable screening of acid-glutaminase activity

In recent reports, it was shown that the Rice test (Rice et al., 1993), a colorimetric assay initially performed on *E. coli*, allows to reliably distinguish amongst *Brucella* strains (and their mutant derivatives) those possessing an active GadB (positive to the Rice test) from those having the relevant gene inactivated by mutations and therefore with an AR2 not functional (negative to the Rice test) (Occhialini et al., 2012; Damiano et al., 2015). The acid assay solution is hypertonic (9% of NaCl), cell-permeabilizing (0.3% of Triton X-100) and unbuffered, so that it allows to detect the GadB activity in bacterial cells because the color of the pH indicator (bromocresol green) in the assay solution turns from yellow to green and then to blue, as protons are consumed (i.e., pH increases) during the decarboxylation of L-Glutamic acid present in the assay solution.

**Figure 2:**
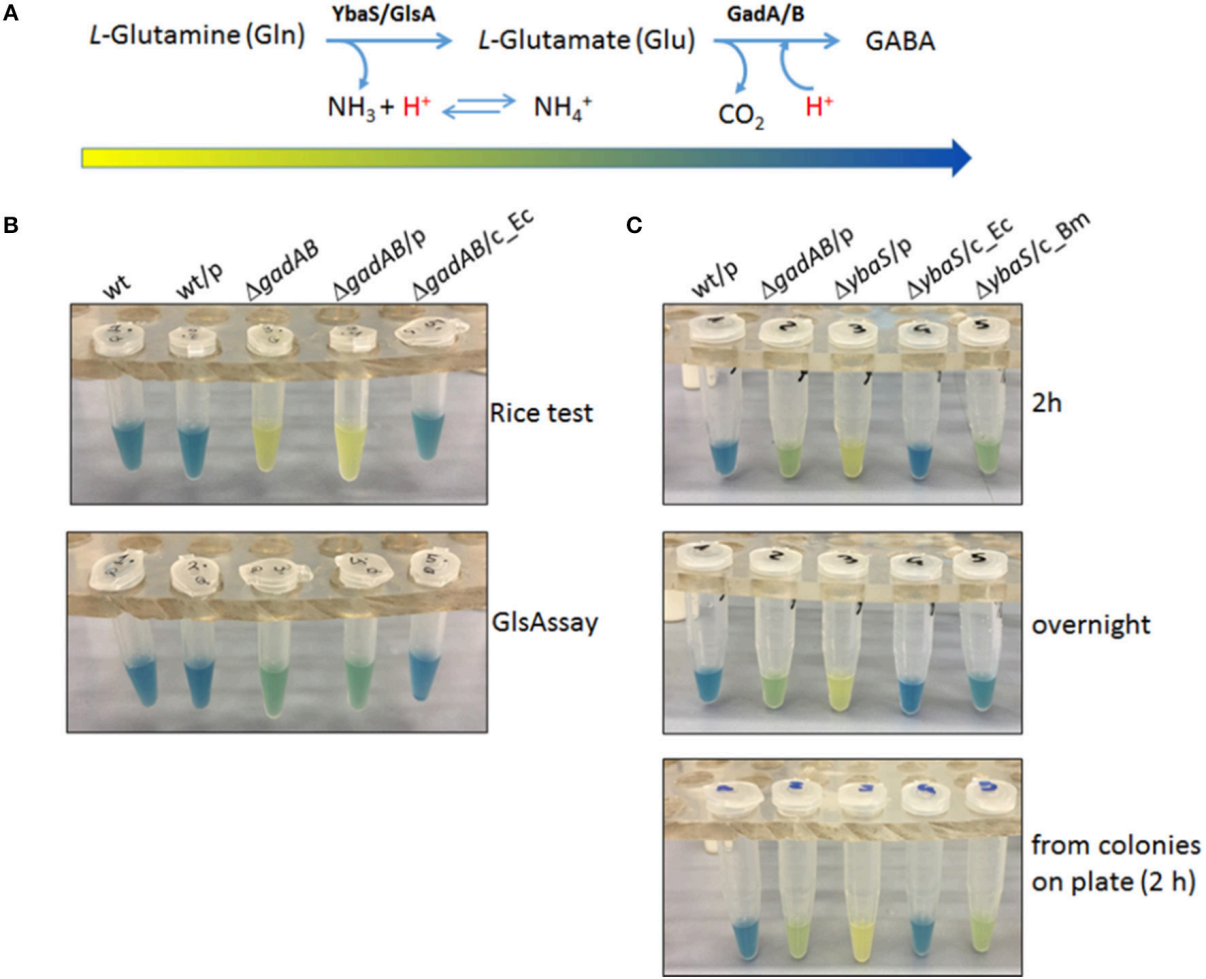
The GlsAssay in *Escherichia coli* K12 MG1655. **(A)** Working hypothesis of the GlsAssay based on the substrates and the enzymes involved. The arrow that begins yellow and ends blue corresponds to the approximate change in color that is expected to occur as the GlsAssay develops, using Gln as starting molecule. **(B)** Preliminary controls. Comparison between the Rice test performed using glutamic acid (upper panel) and the GlsAssay performed using glutamine (lower panel). The strains used were: *E. coli* wild type (wt); wt/pBBR (wt/p); Δ*gadA*-Δ*gadB* (Δ*gadAB*); Δ*gadA*-Δ*gad/*pBBR (Δ*gadAB*/p); Δ*gadA*-Δ*gad/*pBBR_*gadBC* (Δ*gadAB*/c_Ec). The presence of the empty plasmid pBBR1MCS (p) had no effect on the outcome of both tests. The strains were grown for 24 h in LBG pH 5.0, typically used for AR2 assay and Rice test in *E. coli* (Occhialini et al., 2012; Damiano et al., 2015). The GlsAssay was carried out at 37°C overnight. **(C)** Optimized conditions of GlsAssay as described in Materials and Methods (section Rapid Glutaminase Assay (“GlsAssay”) and in Figure S2). The assay was carried out on the strains: wt/pBBR (wt/p); Δ*gadA*-Δ*gadB*/pBBR (Δ*gadAB*/p); Δ*ybaS*/pBBR (Δ*ybaS*/p); Δ*ybaS*/pBBR-*ybaS_Ec* (Δ*ybaS*/c_Ec); Δy*baS*/pBBR-*glsA*_*Bm* (Δ*ybaS*/c_Bm). The incubation time is shown on the right. p, plasmid pBBR1MCS; c, complemented strain with the missing gene.

It was therefore hypothesized that an assay solution as above, but in which *L*-Glutamine (Gln) replaces *L*-Glutamic acid (Glu) would allow to detect the activity of the acid-glutaminase (which is named YbaS in *E. coli* and GlsA in *Brucella* spp. and other bacteria) in a similar way as the Rice test allows the detection of GadB activity. The new assay was named “GlsAssay” (hereafter GlsAssay). According to the working hypothesis (see arrow in Figure 2A), the pH indicator bromocresol green in the unbuffered solution of the GlsAssay should change color upon pH increase due to the formation of ammonium ion (NH${}_{4}^{+}$) from ammonia NH_3_ (which at acidic pH remains in solution) during the deamination of glutamine into glutamate by YbaS/GlsA. Moreover, if the GadB activity is present in the bacterial strain under analysis, the glutamine-derived glutamate should feed GadB, which is also active at acidic pH, to yield GABA and a further increase in pH, as monitored by a change in color into blue (Figure 2A).

The optimal conditions for GlsAssay were: a starting pH of the assays solution in the range 2.8–3.1 (Figure S1A), a density of liquid culture at OD_600_ = 2.0 (0.2–1.0 × 10^9^ cells/ml), and an incubation at 37°C of 2 h (Figure S1B, picture framed in red). At this time point there was a full development of the color which did not change even after an overnight incubation. More cells can be used, i.e., up to twice as much, though this might lead the negative control strain (*E. coli* Δ*ybaS*) to yield a greenish-yellow color, because of the likely alkalinizing effect of too many cells permeabilized in 150 μl of the unbuffered GlsAssay solution (Figure S1B, left panels).

In Figure 2 are summarized the key features of the GlsAssay. In particular, in Figure 2B a comparison of the GlsAssay with the Rice test is provided. This shows that the *E. coli* Δ*gadA*-Δ*gadB* mutant (devoid of glutamate decarboxylase activity) was, as expected, yellow at the Rice test, because this test uses *L*-glutamic acid and therefore cannot detect the glutaminase activity. However, *E. coli* Δ*gadA*-Δ*gadB* in the GlsAssay gave a green color because the presence of glutamine in the GlsAssay solution allows to detect the YbaS activity, which is clearly not affected by the Δ*gadA*-Δ*gadB* mutation. The green color detected in the absence of GadA/B, even after a prolonged incubation, suggested that the pH increase as well as the buffering of the ammonium ion and the glutamate formed during Gln deamination do not allow the solution to go much above pH 4.0–4.5, at which pH the bromocresol green is indeed in the green-absorbing form. In Figure 2C it is shown that *E. coli* Δ*ybaS* was yellow and therefore negative, as hypothesized, and that the color developed with the GlsAssay after 2 h remained yellow even when the incubation was prolonged to an overnight (Figure 2C middle panel).

Overall, it is possible to conclude that the GlsAssay allows to detect the presence of acid-glutaminase activity (samples 3 and 4 in Figure 2B lower panel), clearly not detectable when glutamate is used as substrate (i.e., Rice test; samples 3 and 4 in Figure 2B, upper panel) and gives a negative results (i.e., yellow) when the strain to be assayed does not possess the gene coding for the acid-glutaminase YbaS/GlsA (Δ*ybaS*), even after an overnight incubation (sample 3 in Figure 2C, middle panel). The wild type *E. coli* strain and its Δ*ybaS* derivative strain complemented with a plasmid carrying the *ybaS* gene from *E. coli* gave the expected blue color (samples 1 and 4 in Figure 2C), which was the result of an additive effect of the YbaS and GadA/B activities in these strains. The blue color obtained with these strains is clearly arising from the more effective irreversible proton consumption carried out by GadB and the consumption of glutamate, which result in an increase of the pH to above 5.0, where the bromocresol green is blue-absorbing (Figure 2A).

Finally, it was tested if the GlsAssay could be rendered even faster by using as starting material colonies picked directly from a plate (see section GlsAssay From Colonies on Plate) rather than bacteria from a liquid culture. Indeed Figure 2C (bottom panel) shows that the assay was equally effective regardless of whether the bacteria came from colonies picked on a plate or from a liquid culture. Figure S2 schematically summarizes the steps of the GlsAssay, starting either from colonies on plate or from liquid cultures, and how to interpret the results according to the scheme provided in Figure 2A.

### The GlsAssay allows to screen the best growth conditions for the expression of the acid-glutaminase

**Figure 3:**
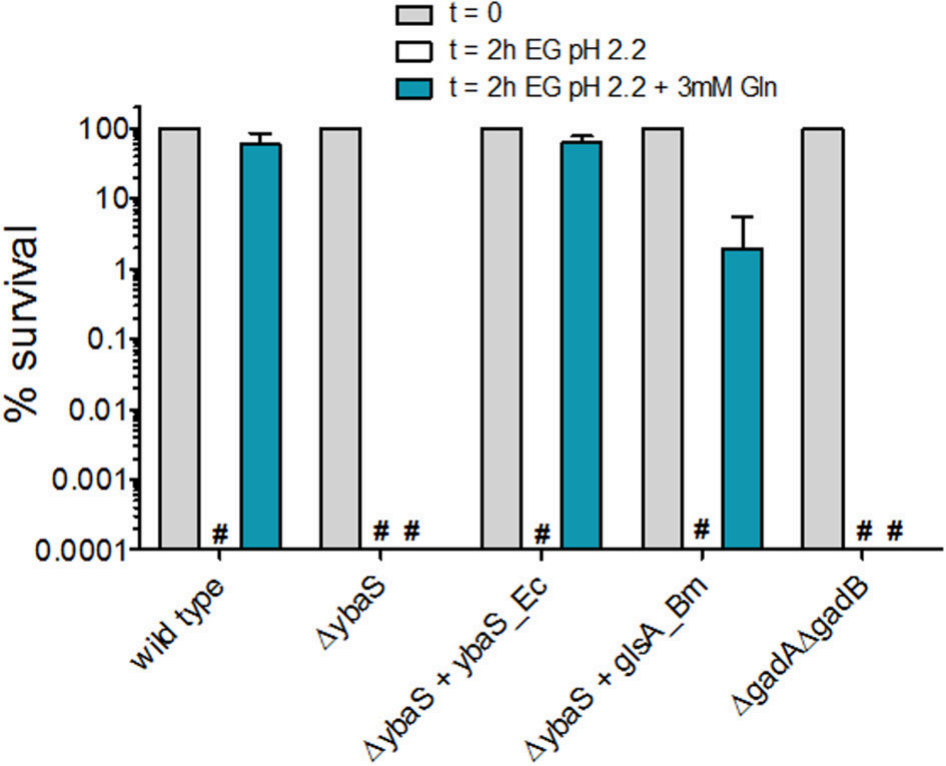
Effect of Gln addition to EG minimal medium at pH 2.2 on the survival of *E. coli* MG1655 and its isogenic mutant strains. Residual viability of stationary-phase cultures of the indicated strains, diluted 1:4000 in EG pH 2.2 and incubated (statically) for 2 h at 37°C. Following the acid challenge, viability was expressed as % CFU/ml on a log_10_ scale, compared to bacteria present at time zero. The data represent the mean (SD) of 3–6 independent experiments. The hashtag indicates no survivors detected. The complemented strains carried the relevant gene cloned into plasmid pBBR1MCS. For consistency, the empty plasmid was also carried by the mutant strains.

As it can be noticed in Figure 2C and Figure S1B, during the setting up of the GlsAssay, the strain *E. coli* Δ*ybaS*/pBBR-*glsA*_*Bm* was also assayed (in the rightmost test tube). This strain was tested to assess for the ability of the *glsA* gene of *Brucella microti* to complement for the mutation in *E. coli* Δ*ybaS*. Indeed *B. microti glsA* was shown to be involved in the AR2_Q of this microorganism and the *E. coli ybaS* gene was demonstrated to complement for the *glsA* mutation in *B. microti* (Freddi et al., 2017). Complementation of *E. coli* Δ*ybaS* with the plasmid pBBR-*glsA*_*Bm* gave different results depending on the medium in which the bacteria were grown prior to the GlsAssay (to detect GlsA activity) and the AR2_Q assay. According to the intensity of the green in *E. coli* Δ*gadA*-Δ*gadB* (sample n. 2 in Figure 2C, Figure S3A), the endogenous acid-glutaminase activity in *E. coli* increased when the pH of the medium in which the bacteria were grown prior to perform the assay was increased (from 5 to 8.0) and when glucose was present. However, while the plasmid carrying *ybaS* from *E. coli* (*ybaS*_*Ec*) could restore the expected phenotype in the GlsAssay (i.e., blue; sample n. 4 in Figure 2C and Figure S1B) and in the AR2_Q assay (Figure 3 and Figure S3B), regardless of the medium tested, the strain carrying the plasmid with the *glsA* gene of *B. microti* (*glsA*_*Bm*) gave positive results only when the bacteria were cultivated in LBG-MOPS, pH 8.0, prior to performing the GlsAssay (sample 5 in Figure 2C vs. Figure S3A) and the AR2_Q assay (Figure 3, Figure S3B). Also, in the latter case the recovery of the AR phenotype in the *E. coli* Δ*ybaS* carrying the *glsA* gene from *B. microti* was only partial, though significant (i.e., more than 5 logs).

Thus, the GlsAssay on the strain *E. coli* Δ*gadA*-Δ*gadB* confirmed that the growth in LBG-MOPS, pH 8.0 was optimal for endogenous YbaS expression and activity, as already reported by Lu et al. (2013). In addition to this, the GlsAssay allowed to demonstrate that the overexpression of the *glsA* gene of *B. microti* could restore the missing activity in the *E. coli* Δ*ybaS* mutant (Figure 2C, Figure S3A) and that this resulted in recovery of the AR2_Q phenotype (Figure 3, Figure S3B) only when the overnight growth of *E. coli* Δ*ybaS*/pBBR-*glsA*_*Bm* was carried out in LBG-MOPS, pH 8.0. This represented a key finding because when using media at lower pH, the recovery of the AR2_Q phenotype turned out to be negative or unsatisfactory (Figure S3B).

Notably, the AR phenotype of the *E. coli* Δ*gadA*-Δ*gadB* mutant in the presence of glutamine was always nil (Figure 3, Figure S3B). Thus, this mutant, though possessing a fully functional *ybaS* gene, is acid-sensitive in the presence of glutamine. This findings points out to the importance of the integrity of the *ybaS* and *gadBC* genes for the full development of a AR2_Q phenotype in *E. coli* and in this respect these results are different from those obtained in *B. microti, L. reuteri* and a pathogenic strain of *E. coli* (Lu et al., 2013; Teixeira et al., 2014; Freddi et al., 2017), where the AR2_Q system (i.e. YbaS/GlsA + GadC) seems to be sufficient to protect from extreme acid stress, i.e., pH ≤ 2.5. The assay conditions, i.e., higher number of cells and higher Gln levels used in pathogenic *E. coli* and *L. reuteri* (Lu et al., 2013; Teixeira et al., 2014), and the GlsA-GadC over-expression in the *B. microti* strain (Freddi et al., 2017) assayed may well explain the discrepancy.

### A quantitative HPLC assay allows to monitor the pH-dependent uptake of glutamine and the export of glutamate and GABA *in vivo*

As far as *E. coli* is concerned, the above observation on the acid-sensitive phenotype of the *E. coli* Δ*gadA*-Δ*gadB* mutant is not fully in line with a previous report (Lu et al., 2013). On the other hand, the GlsAssay in the present report (Figure 2) as well as previous data (Lu et al., 2013) confirmed that YbaS was still active in *E. coli*Δ*gadA*-Δ*gadB*. In order to understand the origin of the acid-sensitive phenotype of *E. coli* Δ*gadA*-Δ*gadB* and to show how Gln uptake is linked to Glu and/or GABA export *in vivo*, a HPLC-based method was developed. This method allowed to quantitatively assay Gln, Glu and GABA in samples (i.e. supernatants) from bacterial cells exposed for 1 h at 37°C to pH 2.5, 3.1, 3.5, and 4.0 in minimal medium EG containing 3 mM Gln.

The details of the protocol used are provided in Materials and Methods (section HPLC Analysis of Glutamine, Glutamate and GABA in Spent Media) and reported schematically in Figure S4. This method allows the quantitation of Gln, Glu, and GABA starting from a few microliters of the acidic medium to which the bacteria were exposed, as reported in section Sample Preparation. Briefly, bacteria (3 × 10^8^ cells, which correspond to at least 10–20 times more cells than in the AR assay in Figure 3) were incubated for 1 h at 37°C in EG medium containing 3 mM Gln at pH 2.5, 3.1, 3.5, and 4.0, respectively. After incubation, an aliquot of samples was kept aside to assay the amino acids by HPLC, while another aliquot was immediately plated to perform a bacterial count that provided information on cell viability (Figure S5).

**Figure 4:**
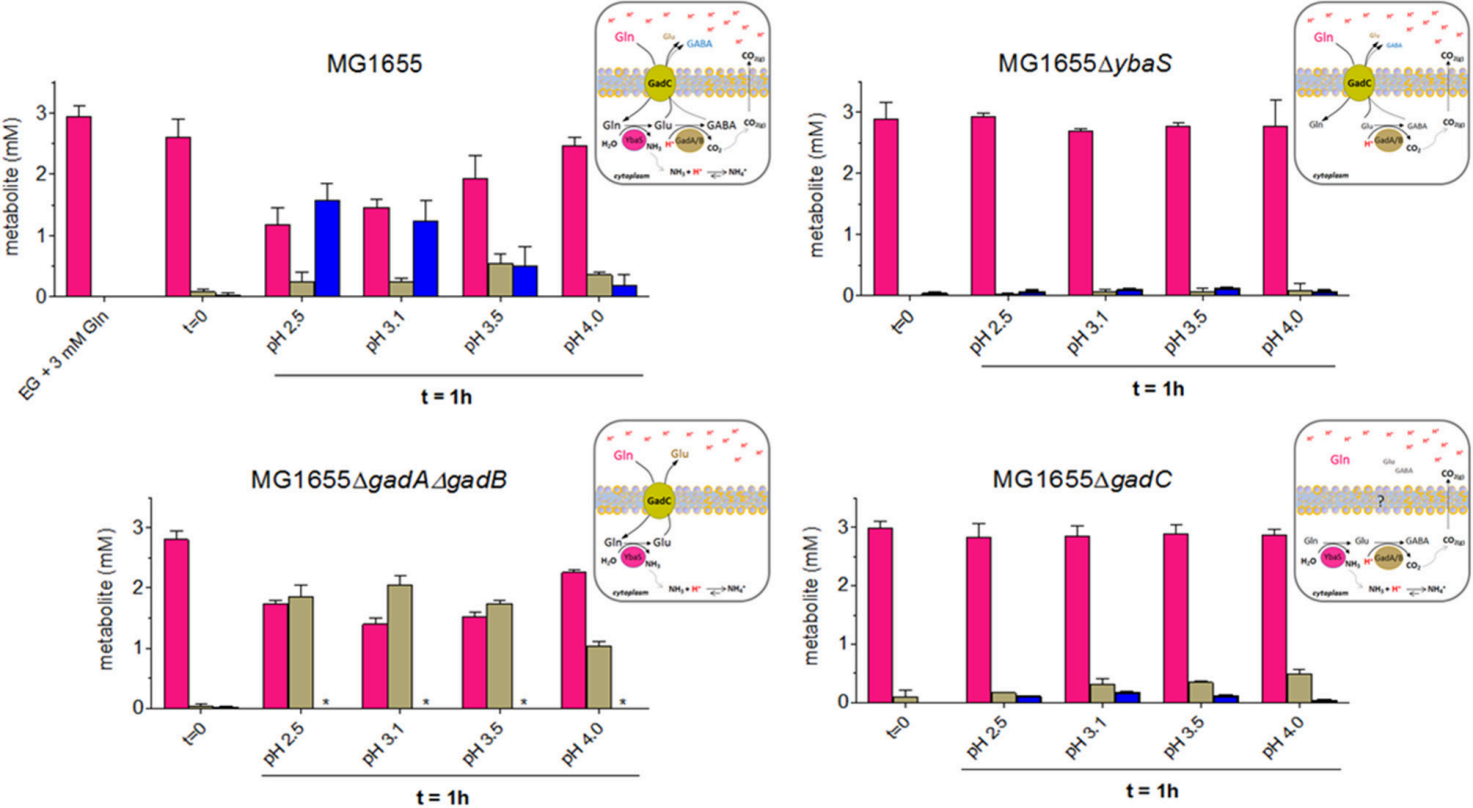
HPLC analysis of Gln, Glu and GABA levels in EG medium at different pHs and from the different mutant strains derived from wild type *E. coli* MG1655. Following the protocol described in Materials and Methods (Section HPLC Analysis of Glutamine, Glutamate and GABA in Spent Media), and represented graphically in Figure S4, bacteria were incubated for 1 h at 37°C. Each spent medium was analyzed by HPLC for glutamine (magenta), glutamate (light dove) and GABA (blue) content. Each panel is accompanied by a schematic representation of the cellular context that provides a possible explanation for the observed findings. ^*^means GABA not detected.

The results of the quantitation using the HPLC, shown in Figure 4, indicated that depending on the pH and the genotype the cells had different behaviors.

The results shown in Figure 4 provided the following indications:

- in *E. coli* wild-type, the cells consumed Gln and released mainly GABA, only at the most acidic pH tested (i.e., 2.5 and 3.1), while the content of Glu in the medium increased in the sample incubated at pH 3.5, where GABA decreases significantly. At pH 4.0 Gln was very little consumed and, accordingly, Glu and GABA levels were low. This observation was in line with the hypothesis that turning off the transport path provided by GadC *in vivo* is more likely to happen in the lower pH range, with respect to what has been reported in the *in vitro* studies employing reconstituted proteoliposomes (Richard and Foster, 2004; Ma et al., 2012).
- in *E. coli* Δ*ybaS*, the cells were unable to use Gln, which was entirely found in the medium, regardless of the pH. Trace amounts of Glu and GABA were detected. A possible explanation for this finding is that some intracellular Glu was converted by GadA/B (still functional in the Δ*ybaS* mutant) into GABA, which was then exported in exchange for some Gln entering in the cell via GadC (which is expected to be functional in the *ybaS* mutant). The observed trace amounts of Glu and GABA are unlikely to originate from cells lysis because the viability (at least in the samples at pH 3.5 and 4.0) was close to 100% after 1 h of incubation (Figure S5) and therefore it is unlikely to be the explanation for the Glu and GABA levels detected in all the samples tested.
- in *E. coli* Δ*gadA*-Δ*gadB*, both decarboxylases are not present, while GadC (Occhialini et al., 2012) and YbaS are functional. The cells used Gln and exported significant amounts of Glu. Maximal Gln consumption was observed at pH 3.1–3.5 (Figure 4, left bottom graph), suggesting that this pH is indeed the optimal external pH for GadC, to export Glu (pI 3.25). Notably under the conditions for the HPLC dosage, survival of *E. coli* Δ*gadA*-Δ*gadB* was observed though full AR was not recovered (Figure S5 vs. Figure 3), thus confirming that the discrepancy between the results in Figure 3 and those published by others (Lu et al., 2013) can be reasonably explained by the starting number of bacteria used in the AR assay.
- in *E. coli* Δ*gadC*, the cells did not import Gln, but some Glu and very little GABA were detected in the medium, though in this case Glu was detected mainly at pH 4.0 with respect to the lower pHs, thus suggesting that another transporter, otherwise silent or less active when GadC is present, was involved in this activity, maybe compensating for the lack of GadC. The observed Glu and traces of GABA are unlikely to originate from cells lysis because the viability (at least in the samples at pH 3.5 and 4.0) was above 70% after 1 h of incubation (Figure S5) and therefore cell death cannot be the explanation for the low levels of Glu and GABA detected in all the samples tested. Furthermore, cell lysis would release YbaS and GadA/B which would consume Gln (just as in the GlsAssay) to GABA. However, GABA was hardly detected, which points to the fact that the transport activity by live cells was indeed measured.

In light of these HPLC data, it was concluded that when Gln was available extracellularly at acidic pH, the *E. coli* wild-type cells preferred to export GABA at pH below 3.1, whereas they exported Glu when exposed to a pH less extreme (3.1–3.5), to finally reduce the export activity of both molecules when the pH was above 4.0. The latter can be most likely attributed to a significantly lower activity of intracellular YbaS and GadA/B (at an external pH of 4 the cytoplasm is expected to be ≥ 6.0) and of GadC at the level of the membrane. The results with the *gadC* mutant provided strong evidence that in the absence of GadC, Gln remained in the medium and was not taken up by the cells; under acidic conditions, GadC is thus the only membrane protein allowing Gln influx. The absence of YbaS had a similar effect, i.e., Gln mostly in the extracellular medium, thus confirming that YbaS is the only glutaminase that allows the deamination of Gln to yield Glu in the acidic pH range. The striking phenotype of the Δ*gadA*-Δ*gadB E. coli* mutant confirmed that GadC is indeed operative in this mutant by importing Gln and exporting Glu, as already suggested by *in vitro* studies using proteoliposomes (Ma et al., 2013; Tsai et al., 2013). However, this did not compensate for the absence of GadA/B, because under the “classical” assay conditions used for measuring the AR phenotype in *E. coli* (Lin et al., 1995, 1996; De Biase et al., 1999) Δ*gadA*-Δ*gadB* showed a strongly acid-sensitive phenotype at pH ≤ 2.5 (Figure 3).

### In the genomes of many enteric bacteria the *glsA* gene is adjacent to the *gadB* and *gadC* genes

The previous reports on the glutamine/acid-glutaminase-dependent AR system (Lu et al., 2013; Teixeira et al., 2014; Freddi et al., 2017) and the results presented above suggested that there is a strong functional link between the AR systems using Gln (AR2_Q) and Glu (AR2). However, the location of the genes *ybaS, glsA*, and *gls3* in the genomes of *E. coli, B. microti*, and *L. reuteri*, respectively, turned out to be different: the gene coding for the acid-glutaminase can be either far from *gadBC* (as in *E. coli*), or immediately downstream the *gadBC* genes with which it constitutes an operon (as in *B. microti*) or preceding the *gadCB* genes, again in a likely operon arrangement (as in *L. reuteri*).

**Figure 5:**
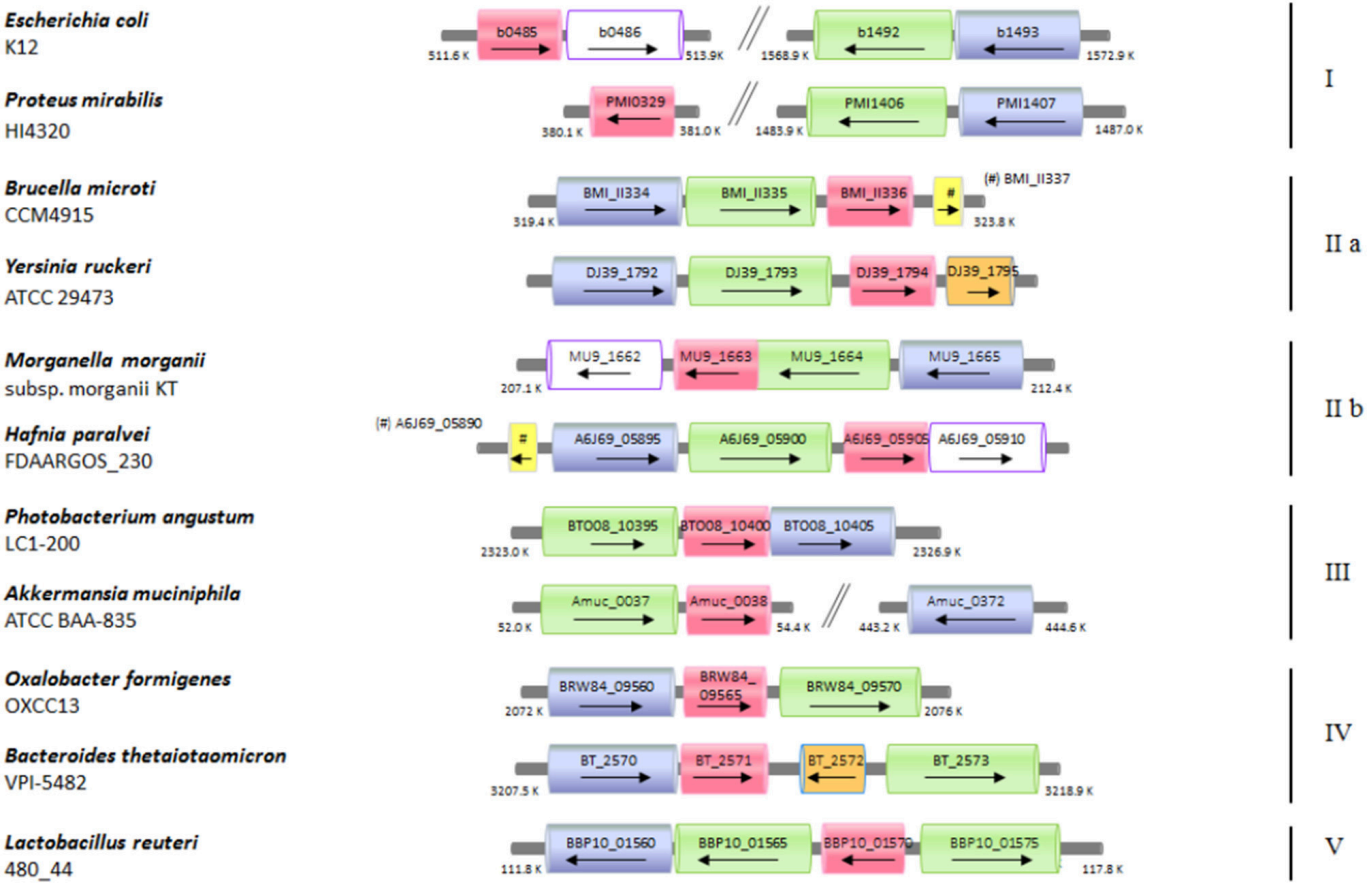
Schematic representation of the distribution of the genes coding for *ybaS*/*glsA, gadB*, and *gadC* in different bacterial genomes. Selected bacterial species and strains, where a potentially functional AR2_Q system occurs, are shown. The arrow lengths and the relative distances are proportional to the gene lengths and distances between adjacent genes, respectively. The corresponding locus tags are shown within each arrow. The homologous genes are represented in different colors: *ybaS/glsA*, in magenta; *gadC*, in green; *gadB*, in blu; *ybaT*, in white with violet contour; *hdeA/B* (periplasmic chaperone) in yellow; putative potassium channel, in orange. The genes with putative functions are dashed in the same color as that of the genes with an assigned function.

The functional link between the glutamine and glutamate AR systems suggested that the arrangement of these genes in *E. coli* could be an exception rather than a rule and that *ybaS*/*glsA* gene more typically is near the *gadB* and *gadC* genes, as in *B. microti* and *L. reuteri*. Moreover, it was previously shown that *gadB* and *gadC* are adjacent in many orally-acquired microorganisms and typically constitute an operon (De Biase and Pennacchietti, 2012). Starting from all the above-mentioned observations and findings, an extensive search for *ybaS*/*glsA* in all those bacteria possessing a *gadBC* operon was carried out. This was performed using the procedure described in section Bioinformatics in Materials and Methods. The results are reported in full in Table S2 and Figure S6, whereas in Figure 5 and Table 1 are provided the key information. Figure 5 in particular shows only eleven representative members out of the over 70 species that constitute the 5 groups (in roman numbers, from I to V) that were identified (Figure S6 and Table S2).

**Table 1:** Bacteria species possessing a potentially functional glutamine-dependent AR system.

Colors of the relevant genes in Figure 5 and Figure S6			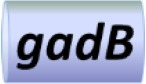		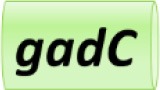		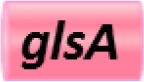	
**^a^Species and Strains**	**^d^Phylum**	**First isolated^e^**	** aa**	***gadB* PATRIC ID or RefSeq locus tag**	** aa**	***gadC* PATRIC ID or RefSeq locus tag**	** aa**	***glsA* PATRIC ID or RefSeq locus tag**
^b^, ^c^*Achromobacter piechaudii* (–) ATCC 43553	PROTEOBAC (beta)	Human	391/73	HMPREF0004_2599/2600	437	HMPREF0004_2601	311	HMPREF0004_2602
*Acidovorax* (–) sp 202149	PROTEOBAC (beta)	Human	459	BMF38_06800	514	BMF38_06795	313	BMF38_08290
*Akkermansia muciniphila* (–) ATCC BAA-835	VERRUCO	Human and animals	466	Amuc_0372	494	Amuc_0037	327	Amuc_0038
*Alistipes shahii* (–) WAL 8301 ^(1)^	BACTEROID	Human	471	ALI_03190	509	ALI_03010	327	ALI_03000
*Bacteroides fragilis* (–) NCTC 9343^(2)^	BACTEROID	Mammals	480	BF0393	411 532	BF0392 BF0487	321	BF0394
*Bacteroides thetaiotaomicron* (–) VPI-5482 ^(2a)^	BACTEROID	Mammals	481	BT2570	570	BT_2573	321	BT_2571
*Barnesiella intestinihominis* (–) YIT 11860	BACTEROID	Human	480	HMPREF9448_00311	504	HMPREF9448_00315	320	HMPREF9448_00312
*Bordetella avium* (–) 197N	PROTEOBAC (beta)	Birds	466	BAV2797	491	BAV2795	312	BAV2794
*Brucella microti* (–) CCM4915 ^(3)^	PROTEOBAC (alpha)	Common vole	464	BMI_II334	485	BMI_II335	317	BMI_II336
*Clostridium perfringens* (+) str. 13	FIRMICUTES	soil	464	CPE2058	472	CPE2060	305	CPE1995
*Desulfovibrio desulfuricans* (–) subsp. desulfuricans str. ATCC 27774	PROTEOBAC (alpha)	Sheep *Ovis aries*	468	Ddes_0045	495 499	Ddes_0046 Ddes_0047	310	Ddes_00484
*Edwardsiella tarda* (–) EIB202 ^(4)^	PROTEOBAC (gamma)	Fish, humans, chickens and other animals	464	ETAE 2868	526	ETAE_2867	295	ETAE_0268
*Enterobacter* (–) sp R4-368	PROTEOBAC (gamma)	Jatropha	461	H650_09405	508	H650_09400	308	H650_03370
*Enterobacteriaceae bacterium* (–) 9_2_54FAA	PROTEOBAC (gamma)	Human	466	HMPref0864_03641	529	HMPref0864_03640	312	HMPref0864_03639
*Enterococcus malodoratus* (+) *ATCC 43197*	FIRMICUTES	Gouda cheese	466 458	I585_01385 I585_04429	503 475 492	I585_01386 I585_02954 I585_04428	312	I585_02953
*Enterovibrio calviensis* (–) 1F_211^(5)^	PROTEOBAC (gamma)	Seawater	459	Figl1190606.3.peg579	518	Figl1190606.3.peg578	313	Figl1190606.3.peg577
*Escherichia albertii* (–) KF1	PROTEOBAC (gamma)	Human	466	EAKF1_ch0011	511	EAKF1_ch0012	310	EAKF1_ch0947
*Escherichia coli* (–) *K12* MG1655 ^(6)^	PROTEOBAC (gamma)	Human	466	b1493	511	b1492	310	b0485
*Escherichia fergusonii* (–) ATCC35469	PROTEOBAC (gamma)	Human	466 466	EFER_2817 EFER_1575	511	EFER_1577	315	EFER_2818
*Eubacterium limosum* (+) KIST612	FIRMICUTES	Sheep	472	ELI_0972	545	ELI_0973	313	ELI_2455
*Fusobacterium nucleatum* (–) subsp. animalis, strain KCOM 1279 ^(7)^	FUSOBACT	Subgingival dental plaque, periimplantitis	459	RN98_06450	479	RN98_06445	304	RN98_03350
*Grimontia indica* (–) AK16	PROTEOBAC (gamma)	Water	459	D515_2780	519	D515_2781	312	D515_2782
*Hafnia paralvei* (–) strain FDAARGOS_230	PROTEOBAC (gamma)	Human	466	A6J69_05895	529	A6J69_05900	312	A6J69_05905
*Izhakiella capsodis* (–) strain N6PO6	PROTEOBAC (gamma)	mirid bug, *Capsodes infuscatus*	466	SAMN05216516_101630	516	SAMN05216516_101631	318	SAMN05216516_101632
*Lactobacillus reuteri* (+) strain 480_44	FIRMICUTES	*Mus musculus*	468	BBP10_01560	510 517	BBP10_01565 BBP10_01575	306	BBP10_01570
*Mesorhizobium soli* (–) strain JCM 19897	PROTEOBAC (alpha)	Forestal soil	464	C7I85_04950	517	C7I85_04945	314	C7I85_04940
*Morganella morganii* (–) subsp. morganii KT	PROTEOBAC (gamma)	Human	460	MU9_1665	493	MU9_1664	309	MU9_1663
*Obesumbacterium proteus* (–) strain DSM2777	PROTEOBAC (gamma)	Feces of wild boar	466	DSM2777_06325	515	DSM2777_06320	312	DSM2777_06315
*Odoribacter splanchnicus* (–) DSM 20712	BACTEROID	Human, abdominal abscess	465	Odosp_1307	538	Odosp_0380	321	Odosp_0379
*Oxalobacter formigenes* (–) *OXCC13* strain OXCC13	PROTEOBAC (beta)	Human	465	BRW84_09560	524	BRW84_09570	316	BRW84_09565
*Parabacteroides merdae* (–) ATCC 43184	BACTEROID	Human	479	PARMER_03646	526	PARMER_03642	321	PARMER_03645
*Paraburkholderia xenovorans* (–) LB400 ^(8)^	PROTEOBAC (beta)	Contaminated soil	461 461	Bxe_A3826 Bxe_C0551	506 506	Bxe_A3825 Bxe_C0552	304	Bxe_B1127
*Photobacterium angustum* (–) LC1-200	PROTEOBAC (gamma)	Seawater	466	BTO08_10405	505	BTO08_10395	314	BTO08_10400
*Photobacterium damselae* (–) subsp. damselae strain KC-Na-1	PROTEOBAC (gamma)	Skin lesions on damselfish	466	CAY62_11315	508	CAY62_11305	319	CAY62_11310
*Proteus mirabilis* (–) HI4320	PROTEOBAC (gamma)	Human	463	PMI1407	517	PMI1407	308	PMI0329
*Providencia alcalifaciens* (–) Dmel2 ^(9)^	PROTEOBAC (gamma)	Fruit fly, *Drosophila melanogaster*	466 391	OO9_16601 OO9_18596	512 518	OO9_16606 OO9_18591	311	OO9_18586
*Pseudomonas psychrophila* (–) strain BS3667 ^(10)^	PROTEOBAC (gamma)	Petroleum sludge	465	SAMN04490201_1375	525	SAMN04490201_1373	314	SAMN04490201_1374
*Serratia fonticola* (–) strain DSM 4576	PROTEOBAC (gamma)	Human	466 466	WN53_13795 WN53_24805	512 512	WN53_13800 WN53_24810	307	WN53_03050
*Shewanella halifaxensis* (–) HAW-EB4	PROTEOBAC (gamma)	Sediment	464	Shal_3043	504	Shal_2708	311	Shal_2709
*Shigella flexneri* (–) 2a str. 301 ^(11)^	PROTEOBAC (gamma)	Human	486 466	SF310_0690 SF301_3206	486 511	SF301_0691 SF301_3205	310	SF301_2702
*Tannerella* (–) sp. 6_1_58FAA_CT1	BACTEROID	Human	480	HMPREF1033_20619	509	HMPREF1033_20622	321	HMPREF1033_20620
*Wohlfahrtiimonas chitiniclastica* (–) SH04	PROTEOBAC (gamma)	Fly, *Chrysomya megacephala*	458	F387_10770	489 479	F387_00701 F387_01771	307	F387_00700
*Yersinia enterocolitica* (–) 8081^(12)^	PROTEOBAC (gamma)	Human	466	YE3693	518	YE3692	313	YE3691
*Yersinia ruckeri* (–) 29473	PROTEOBAC (gamma)	Fish	467	DJ39_1792	531	DJ39_1793	312	DJ39_1794

a The species and strains reported in the list are the most representative. The number near each species links to the list (provided below) of all the genomes where the same gene arrangement has been found. They are: ^(1)^A. indistinctus 17126; A. putredinis DSM 17216; A. finegoldii DMS242 (but missing gadB)–^(2)^B. oleiciplenus YIT 12058; B. massiliensis B84634; B. vulgatus; B. dorei ^(2a)^B. caccae ATCC 43185; B. stercoris ATCC 43183; B. cellulosilyticus WH2; B. intestinalis; B. fluxus–^(3)^B. inopinata BO1; Brucella sp. Br2 09RB8910; B. ceti L2/15; B. pinnipedialis BCCN06-44 – ^(4)^E. ictaluri 93-146–^(5)^E. norvegicus.–^(6)^ E. coli 12264 (O76:H^−^); E. coli 50588 (O8:H^−^); E. coli DEC14D; E. coli E101; E. coli M718; E. coli STEC_94C – ^(7)^Fusobacterium nucleatum subsp. polymorphum strain ChDC F30; Fusobacterium periodonticum strain KCOM 1263–^(8)^ the locus tags are of genes located on different chromosome, as indicated by the lettering A, B, and C preceding each number –^(9)^Providencia burhodogranariea DSM 19968 possesses gadBC and glsA but far apart–^(10)^P. fragi P121–^(11)^S. boydii; S. boydii Sb227; S. dysenteriae 1617; S. dysenteriae 225-75; S. dysenteriae CD_74_112–^(12)^Y. frederiksenii ATCC 33641; Y. intermedia ATCC 29909; Y. kristensenii ATCC 33638; Y. kristensenii ATCC 43969.

b In light gray are shown the species for which the genome sequence is not complete. For this reason in Figure 5 and Figure S6 the position of the genes in the genome (K, in kilobases) is not shown.

c (–) Gram–negative bacterium; (+) Gram–positive bacterium

d BACTEROID, Bacteroidetes; FIRMICUTES, Firmicutes; FUSOBACT, Fusobacteria; PROTEOBAC, Proteobacteria [(alpha), Alphaproteobacteria; (beta), Betaproteobacteria; (gamma), Gammaproteobacteria]; VERRUCO, Verrucomicrobia.

e *The species that are recognized as enteric are shown with a gray background. The coloring of the genes and of the corresponding column in the Table are according to those used in Figure 5 and Figure S6*.

Briefly, it was found that the typical gene arrangements were the following: (1) *ybaS*/*glsA* distant from *gadBC* (group I, which included *E. coli*); (2) *glsA* immediately following *gadBC* (groups IIa, which included *B. microti*, and IIb); (3) *glsA* immediately downstream *gadC*, while *gadB*(*C*) is either downstream or, more frequently, distantly located (group III); (4) *glsA* downstream *gadB*, while *gadC* is either immediately downstream or 1–2 genes downstream (group IV); (5) *glsA* preceeding *gadC*, while *gadB* is close (group V, which included *L. reuteri*).

**Figure 6:**
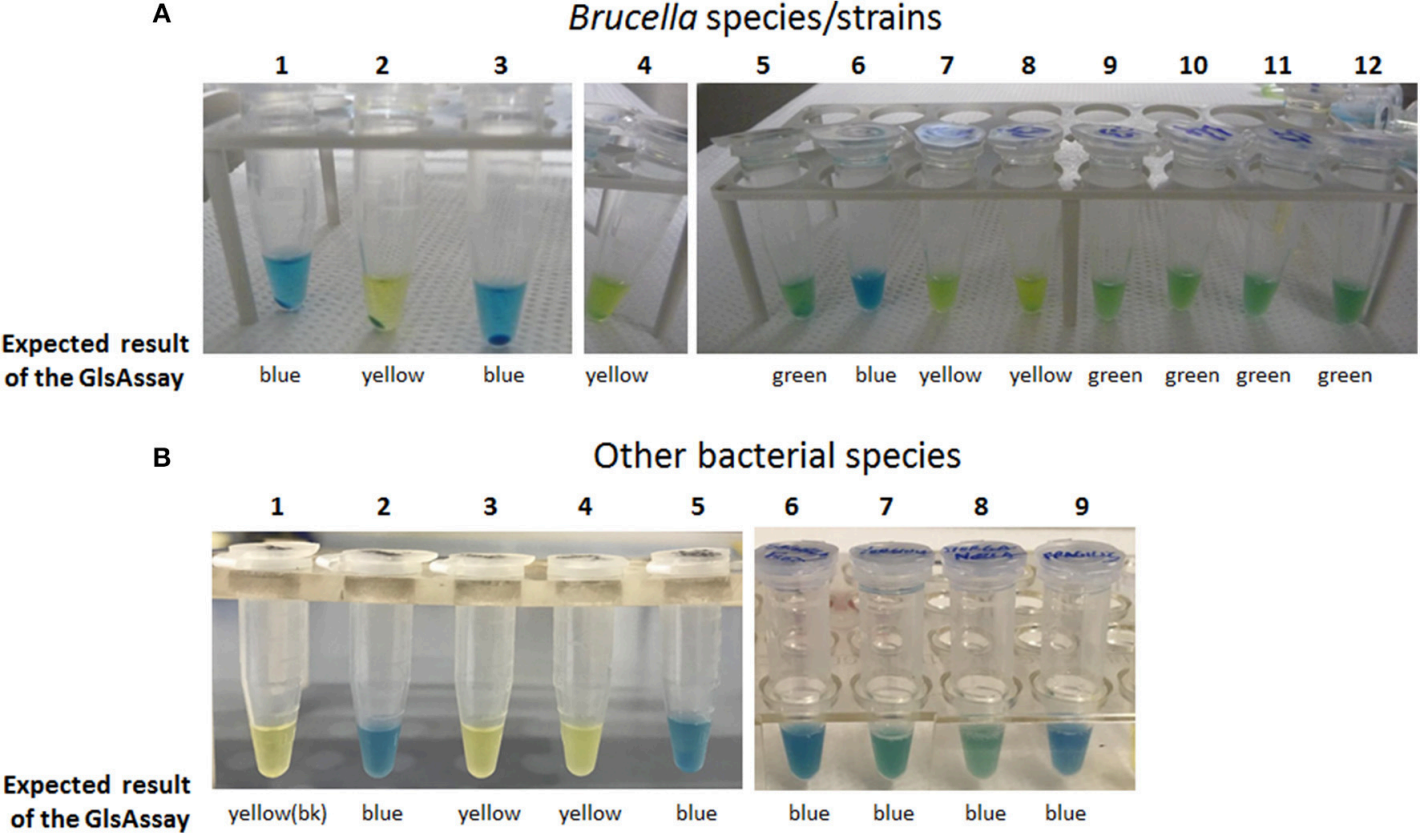
The GlsAssay tested on different bacterial species/strains. **(A)** The GlsAssay was performed starting form colonies on TS plate of the following *Brucella* species/strains: (1) *B. microti* CCM4915 (wild type), (2) *B. microti* CCM4915 Δ*glsA*, (3) *B. microti* CCM4915 Δ*glsA* carrying plasmid pBBR-*glsA*_*Bm*, (4) *B. abortus* ATCC23448 (wild type), (5) *B. abortus* ATCC23448 carrying plasmid pBBR-*glsA*_*Bm*, (6) *B. inopinata* BO1, (7) *B. melitensis* 16M, (8) *B. ovis* ATCC25840, (9) *B. neotomae* 5K33, (10) *B. suis* 1330, (11) *B. suis* S4, (12) *B. canis* RM16/66. **(B)** The GlsAssay was performed starting form liquid cultures (picture on the left) or from colonies (picture on the right) of the following bacterial species*:* (1) GlsAssay solution (blank), (2) *E. coli* MG1655 wild type, (3) *E. coli*Δ*ybaS*, (4) *Salmonella* typhimurium LT2, (5) *Yersinia ruckeri* ATCC29473, (6) *Shigella flexneri* CIP52.36, (7) *Yersinia enterocolitica*, (8) *Morganella morganii* morganii CIP A236, (9) *Bacteroides fragilis* CIP77.16. The incubation was carried out at 37°C for 2–3 h.

Having this list in hands, it was decided to assess the usefulness of the GlsAssay in microorganisms other than *E. coli*, which was used to set up the assay. Using the conditions of the GlsAssay from colonies on plate described in Materials and Methods, many *Brucella* species with the *glsA* gene functional/not functional were tested because their AR2_Q phenotype was already reported and the occurrence of a functional *glsA* gene and the co-presence of functional *gadB* also known (Freddi et al., 2017). In addition *Proteus mirabilis* (group I), *Yersinia ruckeri* and *Yersinia enterocolitica* (group IIa), *Morganella morganii* (group IIb), *Bacteroides fragilis* (group IV) and *Salmonella* typhimurium LT2 (negative at the bioinformatic analysis) were assayed. As shown in Figure 6, all the strains tested gave the expected results at the GlsAssay, as based on the presence of intact/mutated/absent relevant genes. Other strains were tested and the results of the GlsAssay confirmed that they had or not a functional acid-glutaminase, in line with the results of the bioinformatic analysis (data not shown).

## Discussion

The ability to sense and respond to an acid stress is central to ensure that bacteria transiting through the extremely acidic stomach compartment will eventually reach and colonize the gut, in which some areas like the colon are also acidic (De Biase and Pennacchietti, 2012; Lund et al., 2014; De Biase and Lund, 2015). However, the ability to tolerate an high concentration of acids is also of great interest in biotechnology where, through adaptive evolution, microorganisms are rendered more and more tolerant to acids which build up either as part of their same production or as a consequence of the metabolic activities of microbial cell factories involved in the synthesis of valuable chemicals (Raab and Lang, 2011; Liu et al., 2015).

Amongst the most efficient acid resistance (AR) mechanisms in bacteria, the amino acid-dependent systems are quite widespread in Gram-negative and Gram-positive bacteria, including pathogenic ones (De Biase and Pennacchietti, 2012; Feehily and Karatzas, 2013; Lund et al., 2014; De Biase and Lund, 2015). In addition to the glutamate- and arginine-dependent AR systems (also named AR2 and AR3, respectively), more recently the glutamine-dependent AR system (AR2_Q) has received attention, because it was found to be functional not only in *E. coli*, but also in some *Brucella* species isolated from the environment and in the wildlife and in *L. reuteri* (Lu et al., 2013; Teixeira et al., 2014; Freddi et al., 2017). This system is as effective as the AR2, with which it shares the GadC antiporter, which is therefore a key structural element of both systems. Notably, as compared to glutamate, glutamine is an amino acid more readily available in the free form in various food sources as well as in the human body, and represents the major form through which nitrogen is transported in an organic form between cells (Lu et al., 2013; Xiao et al., 2016; Kim and Kim, 2017).

In addition to GadC, a key structural component of the AR2_Q is the enzyme glutaminase YbaS (Lu et al., 2013). In *E. coli* there are two glutaminases, one (YbaS) active in the acidic pH range (i.e., pH 4–6) and one (YneH) active in the neutral-alkaline pH range (i.e., 6.0–8). Notably, the gene coding for GlsA, the *Brucella* homolog of YbaS, is located immediately downstream the *gadBC* genes of the AR2, with which it constitutes an operon together with the downstream *hdeA* gene (coding for a periplasmic chaperone), also involved in AR in *E. coli* (Kern et al., 2007; Tapley et al., 2010; Hong et al., 2012).

Herein, a further characterization of the mechanism of action of the AR2_Q system was undertaken with multiple aims. First, to develop qualitative and quantitative assays as convenient alternatives to those currently used, and second to dissect the pH-dependent transport mechanism oparated by GadC. Finally, through an extensive bioinformatic analysis it was attempted to establish the distribution of AR2_Q in bacteria.

The qualitative assay was named GlsAssay: it is an inexpensive, simple to perform, fast, reliable and sensitive colorimetric assay that allows to test for the expression and functionality of the *ybaS*/*gls*A gene product under acidic conditions and to detect if also GadB (i.e., a component of the AR2) is present and active. This is very easily assessed by observing whether the color developed with the assay remains green (even after a prolonged incubation), which indicates that only the YbaS/GlsA enzyme is present and active, or turns into blue, which points to an additive effect of the proton-consuming activity of GadB, the enzyme glutamate decarboxylase that works under acidic pH conditions too (Rice et al., 1993; Occhialini et al., 2012; Damiano et al., 2015). The results reported herein clearly show that in a physiological background the GlsAssay allows the detection of the acid-glutaminase as well as the co-occurrence of the decarboxylation of glutamate, thus suggesting that intracellularly YbaS/GlsA and GadB work in concert in the bacterial species under analysis.

Even though very informative, the GlsAssay does not provide any information on the *in vivo* uptake of glutamine and which are the molecules released in the extracellular medium (glutamate, GABA or both) depending on the pH of the imposed acid stress. The reports published to date (Lu et al., 2013; Teixeira et al., 2014; Freddi et al., 2017) have dealt only marginally with this point, which is a key point to understand, because the bacterial response to acidic pH involves a number of acid-protecting system, each working at the best within a specific pH range. In other words, the previous reports suggest that in *E. coli* the amino acid glutamine is converted into GABA (via GadA/B activity), but there was no attempt to link this to the pH of the extracellular medium and to quantify the exported substrate(s). On the other hand, we know that GadC, when reconstituted into proteoliposomes, is indeed capable of exporting either glutamate or GABA in exchange for glutamine (Ma et al., 2012, 2013; Tsai et al., 2013). Thus, to evaluate if glutamine is effectively imported by GadC and exported as glutamate and/or GABA, it was necessary to develop an assay that allowed to measure the extracellular levels of these three amino acids at the same time. It should also be reminded that the glutaminase activity was previously detected both *in vivo* and *in vitro* by measuring ammonia production either directly (i.e., using an ammonium ion selective electrode meter) or indirectly (i.e., through a coupled assay using glutamate dehydrogenase) (Brown et al., 2008; Lu et al., 2013), but without any mention on the fate of glutamate arising from glutamine deamination. The results obtained in this work indicate that the AR2 systems (i.e., AR2_Q and AR2) are extremely versatile and that, when Gln is provided extracellularly, GadC exports either GABA, as result of the intracellular activity of the glutaminase YbaS/GlsA and the decarboxylase GadA/B, or glutamate, when only Ybas/GlsA is operative, or both, depending on the acid stress to which the cells are exposed. Overall, the extracellular levels of Glu and GABA reflect the intracellular activity of the two enzymes: GABA is the major product when the acid stress imposed is harsh (pH 2.5), whereas glutamate increases when the extracellular pH is above 3.0. At an extracellular pH > 4.0, the levels of both molecules are low and this clearly reflects the decrease of the intracellular activity of the two enzymes as well as the turning off of the transport channel of GadC, in line with previous studies (Richard and Foster, 2004; Ma et al., 2012). Therefore, this work shows for the first time the pH-dependent turning off of GadC *in vivo* in the pH range 2.5–4.0 and how this membrane protein provides the key path through which GABA and/or Glu are exported in the medium, depending on the extracellular pH, when Gln is supplied. At pH > 4.0 other systems and membrane proteins are expected (and known) to work (Kanjee and Houry, 2013; Lund et al., 2014; Tramonti et al., 2017).

One of the key findings of this work is that the *glsA* gene in many bacteria is strongly associated with the *gadB* and *gadC* genes. A previous study highlighted that *gadB* and *gadC* lie next to/near each other (De Biase and Pennacchietti, 2012) and a recently published computational study confirmed the correctness of those observations (Bradley et al., 2018). In the present work, for the first time, this association is shown to be extended to the *glsA* gene too. Indeed, in four out of the five groups shown in Figure 5 and Figure S6, the *glsA* gene was typically found close to both *gadB* and *gadC* or at least to one of them. This observation comes from the scrutiny of over 1,500 genomes and opens very interesting perspectives, in particular with regards to the relevance of the AR2_Q system (i.e., glutamine-dependent), as compared to the much more characterized AR2 system (glutamate-dependent). This hypothesis, supported by the results of the GlsAssay performed on several strains (Figure 6), needs to be confirmed with more detailed studies aimed at demonstrating the occurrence of a glutamine/glutaminase-dependent AR system in the bacterial species listed in Table 1, many of which are of great interest. Nonetheless, the results of the bioinformatic analysis (Table 1, Table S2, and Figure 5, Figure S6), do provide a strong indication that the glutamine-dependent AR system is likely to occur and be operative in many bacterial species. With the exception of the Gram-positive *Clostridium perfringens* and *Eubacterium limosum* in group I, *Enterococcus malodoratus* in group III, and *Lactobacillus reuteri* in group V, all the other bacterial species belong to the Gram-negatives. Moreover, most of them (70%) are enteric (with an equal distribution between pathogenic and beneficial bacteria), with some notable exceptions of bacteria isolated from soil or seawater (Table 1). This points to a prominent role of the glutamine-dependent AR system in the physiology of bacteria in the gut and in providing an effective mechanism to protect from the extreme acid stress encountered during the transit through the stomach before reaching the gut (Giannella et al., 1972; Martinsen et al., 2005; Beasley et al., 2015). Most of the species belong to the phyla Proteobacteria (approximately 70%) and Bacteroidetes (approximately 20%), with the remainder including four species belonging to the phylum Firmicutes (that also represent the Gram-positives), two species belonging to Fusobacteria and one species belonging to Verrucomicrobia (*Akkermansia muciniphila*). A preliminary observation, restricted to nine genera belonging to the *Enterobacteriaceae* (Djoko et al., 2017), is also in line with the more comprehensive analysis presented in this work.

A notable finding of the bioinformatic analysis is that some genes in one or more groups are frequently found associated to *glsA, gadB*, and *gadC*. These include: the homologs of *E. coli hdeA* and *ybaT* genes, and a gene coding for a putative voltage gated-potassium channel.

The *hdeA* gene was already known to be involved in protection from acid stress in Gram-negative bacteria as it encodes a periplasmic acid-induced chaperone (Kern et al., 2007; Tapley et al., 2010; Hong et al., 2012). This gene was found adjacent to or in between the genes of interest in some members of group II (*Brucella microti, Enterobacteriaceae bacterium*, and *Hafnia paralvei*) and in one member of group III (*Bordetella avium*).

The *ybaT* gene is encountered in some members of group I (i.e., *Escherichia coli, E. albertii, Shigella flexneri, S. boydii, S. dysenteriae*), whereas it is strongly associated to the other three genes in members of group IIb. The present as well as a previous work (Lu et al., 2013) have shown that GadC is the key membrane protein involved in the import of glutamine under acid stress. Also, the substrate specificity of the *ybaT* gene product still needs to be established conclusively, though *ybaT* does not seem to code for a membrane protein playing a key role in AR2_Q. A recent report suggests that the increased expression of *ybaS* and *ybaT* during a copper stress under acidic pH and in the presence of Gln, may counteract the inhibition of the GOGAT enzyme (i.e., glutamate synthase) which occurs under these conditions, thereby overcoming the block of Glu synthesis through this important enzyme (Djoko et al., 2017). Overall, the role of the *ybaT* gene product is still elusive and requires further investigations.

Finally, a gene coding for a “putative” voltage gated-potassium channel was frequently found in some bacterial species, mostly belonging to the phylum Bacteroidetes. In particular *Odoribacter splanchnicus* (in group III), most of the members of group IV, except for *Oxalobacter formigens*, and *Yersinia ruckeri* (in group IIa). These proteins are K^+^-selective and assigned to the Voltage-gated Ion Channel (VIC) Superfamily. A clear function in AR was never reported for these proteins, however the studies on GadC using proteoliposomes and those on the AR2_Q system in *L. reuteri* do point to a role of potassium entry in positively affecting the antiport activity in GadC and AR in general (Ma et al., 2013; Tsai et al., 2013; Teixeira et al., 2014). The role of potassium channels in bacteria is largely unknown, however it is well known that K^+^ is an important cellular cation, for which glutamate represents the major counterion (McLaggan et al., 1994) and that extracellular K^+^ (together with Na^+^) strongly stimulates the activation of the EvgS sensor kinase of the two component system EvgAS that confers acid resistance to *E. coli* (Eguchi and Utsumi, 2014). A strong link between acidic pH in the activity of potassium channels was demonstrated to be physiologically important in *Corynebacterium glutamicum*, where bacteria respond to a decrease in extracellular pH by decreasing the membrane potential through the influx of K^+^ via the ClgK channel, which contributes to maintaining the electrochemical membrane potential constant (Follmann et al., 2009). More recently, the role of acidic pH in the activity of potassium channels was elegantly shown in a structural study on KcsA (a K^+^ channel from *Streptomyces lividans*) (Tilegenova et al., 2017). In general, the literature on bacterial potassium channels is scarce however, the bioinformatic analysis carried out in the present work suggests that there is much more to be understood on the link between acid stress and K^+^ transport in bacteria, in particular in Bacteroidetes that are common inhabitants of the human gut.

Overall, the bioinformatic search for the co-occurrence of the *glsA* gene and *gadBC* genes (regardless if distantly located or adjacent) failed to identify a significant number of Gram–positive bacteria, i.e., only 5 out of the over 70 species included in Table 1, some of which are listed in the footnotes to avoid redundancy. The reason for this is still unclear and will need further studies.

In conclusion, a rapid method for the detection of the glutaminase activity in bacteria was developed in this work. This method can easily find utilization in routine analysis in laboratories and be used for phenotypic screening. Moreover, the HPLC-based analysis provides a tool for studying the pH-dependent activity of GadC in different bacteria. Finally, the bioinformatic study strongly supports of the widespread occurrence of the glutamine/glutaminase-dependent AR system in many bacterial species, including those that are part of the human gut microbiome. This latter finding is particularly important taking into account that Gln is the most abundant amino acid in systemic circulation (0.6 mM) and that it represents an important microbial metabolite in the distal gut (Matsumoto et al., 2012; Mariño et al., 2017).

## Author contributions

DB designed the study. EP, CD, LF, and AO carried out the experiments. EP and DB analyzed the data and performed the bioinformatic analysis. DB and EP drafted the manuscript. All authors contributed to the critical discussion of the results and to the final drafting of the manuscript. The manuscript in the present version was read and approved by all the authors.

### Conflict of interest statement

The authors declare that the research was conducted in the absence of any commercial or financial relationships that could be construed as a potential conflict of interest.
