# Supplementary material for: The Glutaminase-Dependent Acid Resistance System: Qualitative and Quantitative Assays and Analysis of Its Distribution in Enteric Bacteria
Source: Front Microbiol. 2018 Nov 15;9:2869. doi: 10.3389/fmicb.2018.02869 (PMC6250119; doi:10.3389/fmicb.2018.02869)
Supplement: Supplementary file 1 [file Table_1.PDF]

Supplementary Material

The glutaminase-dependent acid resistance system: qualitative and quantitative assays and analysis of its distribution in enteric bacteria

Eugenia Pennacchietti [1], Chiara D'Alonzo[1], Luca Freddi [2], Alessandra Occhialini[2], Daniela De Biase[1*]

*** Correspondence:** Daniela De Biase: daniela.debiase@uniroma1.it

Table S1. Bacterial strains, plasmids and oligonucleotides used in this study.

Bacterial strains and derivatives	Relevant Genotype/ information	Reference/source
***E.coli* MG155**		
MG1655	F^{-} λ^{--} *rph*$^{-1}$	GSC
MG1655/pBBR	F^{-} λ^{--} *rph*$^{-1}$ carrying plasmid pBBR1MCS	(Occhialini et al., 2012)
MG1655Δ*gadA*Δ*gadB*	MG1655 *gadAgadB*::Kan^{R}	(Occhialini et al., 2012)
MG1655Δ*ybaS*	MG1655 *ybaS*::Kan^{R}	This work
MG1655Δ*gadC*	MG1655 *gadC*::Kan^{R}	(Occhialini et al., 2012)
MG1655Δ*gadA*Δ*gadB/*pBBR	MG1655 *gadAgadB*::Kan^{R} carrying pBBR1MCS	(Occhialini et al., 2012)
MG1655Δ*gadA*Δ*gadB/*pBBR-*gadBC_Ec*	MG1655 *gadAgadB*::Kan^{R} carrying pBBR1MCS-*gadBC_ Ec*	(Occhialini et al., 2012)
MG1655Δ*ybaS/*pBBR	MG1655 *ybaS*::Kan^{R} carrying pBBR1MCS	This work
MG1655Δ*ybaS* /pBBR-*ybaS_Ec*(long)	MG1655 *ybaS*::Kan^{R} carrying pBBR1MCS-*ybaS_Ec*(long)	This work
MG1655Δ*ybaS* /pBBR-*ybaS_Ec*	MG1655 *ybaS*::Kan^{R} carrying pBBR1MCS-*ybaS_Ec*	This work
MG1655Δ*ybaS* /pBBR-*glsA_Bm*	MG1655 *ybaS*::Kan^{R} carrying pBBR1MCS-*glsA_Bm*	This work
MG1655Δ*gadC/*pBBR	MG1655 *gadC*::Kan^{R} carrying pBBR1MCS	(Occhialini et al., 2012)
***Brucella* spp.**		
B. microti CCM4915	Wild type	CCM4915 /BCCN07-01
B. microti Δ*glsA*	*B. microti* CCM4915 Δ*glsA*	(Freddi et al., 2017)
B. microti Δ*glsA/*pBBR *glsA_Bm*	*B. microti* CCM4915 Δ*glsA* carrying plasmid pBBR-*glsA_Bm*	(Freddi et al., 2017)
B. abortus 544	Wild type	ATCC23448 (S1)
*B. abortus/*pBBR-*glsA_Bm*	*B. abortus* 544 carrying plasmid pBBR-*glsA_Bm*	(Freddi et al., 2017)
B. canis RM16/66	Wild type	ATCC 23365
B. inopinata BO1	Wild type	CDC San.Ak.BW
B. melitensis 16M	Wild type	ATCC23456
B. neotomae 5K33	Wild type	ATCC23459
B. ovis 63/290	Wild type	ATCC25840
B. suis 1330, bv1	Wild type	ATCC23444
B. suis, S4	Wild type	ATCC23447
Bacteroides fragilis	Wild type	**CIP77.16
Morganella morganii	Wild type subspecies morganii	CIPA236
Salmonella typhimurium	LT2(SF1) Attenuated	(De Biase et al., 1999)
Shigella flexneri	Wild type	CIP52.36

Yersinia enterocolitica	Wild type	Clinical isolate
Yersinia ruckeri	Wild type	ATCC29473
**Plasmids**	**Relevant Genotype/ information**	**Reference/source**
pBBR1MCS	Expression plasmid (4707 bp): *lac*, T3 and T7 promoters, CAT/Cam^R.	(Kovach et al., 1994)
pBBR-*ybaS_Ec*(long)	Plasmid pBBR1MCS carrying a 1121 bp synthetic sequence corresponding to the *E. coli* region encompassing the 933-bp *ybaS* ORF, the 156-bp upstream region of its indigenous promoter, the 20 bp at its 3'end, and additional 6 bp (at the 5'-end) and 6 bp (at the 3'- end) in order to include XhoI and XbaI restriction sites, respectively, for directional cloning into pBBR1MCS.	(Freddi et al., 2017)
pBBR-*ybaS_Ec*	Plasmid pBBR-*ybaS_Ec*(long) in which the 110 bp of the *ybaS* promoter region were removed by digestion with XhoI and BamHI	This work
pBBR-*glsA_Bm*	pBBR1MCS containing the 1768 bp region coding for *glsA* from *B. microti* reference type strain CCM4915	(Freddi et al., 2017)
**Oligonucleotides**		
Δ*ybaS*_for(P1)*	5'-ATGTTAGATGCAAACAAATTACAGCAGGCAGTGGATCA*TGTAGGCTGGAGCTGCTTC*-3'	
Δ*ybaS*_rev(P4)*	5'-TTTTTGACCGCGAACACTGTTGCCATCTTCGTCCAGC*ATTCCGGGGATCCGTCGACC*-3'	
k1	5'-CAGTCATAGCCGAATAGCCT-3'	
*ybaS*_rev	5'-GGTTATAGCCGAGTTGCTT-3'	

*The nucleotide sequences annealing upstream (P1) and downstream (P4) of the template plasmid pKD13 are shown in italics.
** CIP, Collection Institute Pasteur

References

De Biase, D., Tramonti, A., Bossa, F., and Visca, P. (1999). The response to stationary-phase stress conditions in *Escherichia coli*: role and regulation of the glutamic acid decarboxylase system. *Mol Microbiol* 32(6)**,** 1198-1211.

Freddi, L., Damiano, M.A., Chaloin, L., Pennacchietti, E., Al Dahouk, S., Kohler, S., et al. (2017). The Glutaminase-Dependent System Confers Extreme Acid Resistance to New Species and Atypical Strains of *Brucella*. *Front Microbiol* 8**,** 2236. doi: 10.3389/fmicb.2017.02236.

Kovach, M.E., Phillips, R.W., Elzer, P.H., Roop, R.M., 2nd, and Peterson, K.M. (1994). pBBR1MCS: a broad-host-range cloning vector. *Biotechniques* 16(5)**,** 800-802.

Occhialini, A., Jimenez de Bagues, M.P., Saadeh, B., Bastianelli, D., Hanna, N., De Biase, D., et al. (2012). The glutamic acid decarboxylase system of the new species *Brucella microti* contributes to its acid resistance and to oral infection of mice. *J Infect Dis* 206(9)**,** 1424-1432. doi: 10.1093/infdis/jis522.
