# Supplementary material for: The Glutaminase-Dependent Acid Resistance System: Qualitative and Quantitative Assays and Analysis of Its Distribution in Enteric Bacteria
Source: Front Microbiol. 2018 Nov 15;9:2869. doi: 10.3389/fmicb.2018.02869 (PMC6250119; doi:10.3389/fmicb.2018.02869)
Supplement: Supplementary file 2 [file Table_2.PDF]

Supplementary Material

The glutaminase-dependent acid resistance system: qualitative and quantitative assays and analysis of its distribution in enteric bacteria

Eugenia Pennacchietti [1], Chiara D'Alonzo[1], Luca Freddi [2], Alessandra Occhialini[2], Daniela De Biase[1*]

*** Correspondence:** Daniela De Biase: daniela.debiase@uniroma1.it

Table S2. Bacteria possessing a potentially functional glutamine-dependent AR system.

Colors of the relevant genes in Fig. 5 and Fig. S6			gadB		gadC		glsA	
**[a] Species and Strains**	**[d] RefSeq**	**First isolated**	**aa**	***gadB* PATRIC ID or RefSeq locus tag**	**aa**	***gadC* PATRIC ID or RefSeq locus tag**	**aa**	***glsA* PATRIC ID or RefSeq locus tag**
[b, c] ***Achromobacter piechaudii*** (-)<br>ATCC 43553	NZ_ADMS00000000	Human	391/73	HMPREF0004_2599/2600	437	HMPREF0004_2601	311	HMPREF0004_2602
Acidovorax (-)<br>sp 202149	na	Human	459	BMF38_06800	514	BMF38_06795	313	BMF38_08290
Akkermansia muciniphila (-)<br>ATCC BAA-835	NC_010655.1	Human and animals	466	Amuc_0372	494	Amuc_0037	327	Amuc_0038
Alistipes shahii (-)<br>WAL 8301 [(1)]	NC_021030.1	Human	471	ALI_03190	509	ALI_03010	327	ALI_03000
Bacteroides fragilis (-)<br>NCTC 9343[(2)]	NC_003228.3	Mammals	480	BF0393	411<br>532	BF0392<br>BF0487	321<br>826<br>836	BF0394<br>BF0297 [#]<br>BF0285 [#]
Bacteroides thetaiotaomicron (-)<br>VPI-5482 [(2a)]	NC_004663.1	Mammals	481	BT2570	570	BT_2573	321<br>840<br>174<br>172<br>171<br>837<br>825	BT_2571<br>BT_3477 [#]<br>BT_3481 [#]<br>BT_3491 [#]<br>BT_3503 [#]<br>BT_3512 [#]<br>BT_3526 [#]

							965	BT_3546 [#]
Barnesiella intestinihominis (-)<br>YIT 11860	NZ_ADLE00000000.1	Human	480<br>588	HMPREF9448_00311<br>HMPREF9448_01161 [#]	504	HMPREF9448_00315	320<br>839	HMPREF9448_00312<br>HMPREF9448_02291 [#]
Bordetella avium (-)<br>197N	NC_010645.1	Birds	466	BAV2797	491	BAV2795	312	BAV2794
Brucella microti (-)<br>CCM4915 [(3)]	NC_013118.1	Common vole	464	BMI_II334	485	BMI_II335	317	BMI_II336
Clostridium perfringens (+)<br>str. 13	NC_003366.1	soil	464	CPE2058	472	CPE2060	305<br>307	CPE1995<br>CPE0591[#]
Desulfovibrio desulfuricans (-)<br>subsp. desulfuricans<br>str. ATCC 27774	NC_011883.1	Sheep, *Ovis aries*	468	Ddes_0045	495<br>499	Ddes_0046<br>Ddes_0047	310	Ddes_00484
Edwardsiella tarda (-)<br>EIB202 [(4)]	NC_013508.1	Fish, humans, chickens and other animals	464<br>570	ETAE 2868<br>ETEA_0786[#]	526<br>484<br>459	ETAE_2867<br>ETAE_0784 [#]<br>ETAE_0785 [#]	295<br>311	ETAE_0268<br>ETAE_2866[#]
Enterobacter (-)<br>sp R4-368	NC_021500.1	Jatropha	461	H650_09405	508	H650_09400	308	H650_03370
Enterobacteriaceae bacterium (-)<br>9_2_54FAA	NZ_ADCU00000000.2	Human	466	HMPref0864_03641	529	HMPref0864_03640	312<br>308	HMPref0864_03639<br>HMPref0864_02653 [#]
Enterococcus malodoratus (+)<br>ATCC 43197	NZ_ASWA00000000.1	Gouda cheese	466<br>466<br>458	I585_01385<br>I585_02041[#]<br>I585_04429	503<br>475<br>492	I585_01386<br>I585_02954<br>I585_04428	312	I585_02953
Enterovibrio calviensis (-)	na	Seawater	459	Figl1190606.3.peg579	518	Figl1190606.3.peg578	313	Figl1190606.3.peg577

1F_211 [5]			546	Fig1190606.3peg1910 #			306	Figl1190606.3.peg1080#
Escherichia albertii (-)<br>KF1	NZ_CP007025.1	Human	466<br>466	EAKF1_ch0011<br>EAKF1_ch2433 #	511	EAKF1_ch0012	310<br>308	EAKF1_ch0947<br>EAKF1_ch4524#
Escherichia coli (-)<br>K12 MG1655 [6]	NC_000913.3	Human	466<br>466	b1493<br>b3517 #	511	b1492	310<br>308	b0485<br>b1524 #
Escherichia fergusonii (-)<br>ATCC35469	NC_011740.1	Human	466<br>466	EFER_2817<br>EFER_1575	511	EFER_1577	315<br>308	EFER_2818<br>EFER_1550 #
Eubacterium limosum (+)<br>KIST612	NC_014624.2	Sheep	472	ELI_0972	545<br>475	ELI_0973<br>ELI_3345 #	313	ELI_2455
Fusobacterium nucleatum (-) subsp. animalis<br>strain KCOM 1279 [7]	NZ_CP012713.1	Subgingival dental plaque, periimplantitis	459	RN98_06450	479	RN98_06445	304	RN98_03350
Grimontia indica (-)<br>AK16	NZ_ANFM00000000.2	Water	459<br>547	D515_2780<br>D515_1413 #	519	D515_2781	312<br>306	D515_2782<br>D515_0095 #
Hafnia paralvei (-)<br>strain FDAARGOS_230	na	Human	466	A6J69_05895	529	A6J69_05900	312<br>308	A6J69_05905<br>A6J69_02780*
Izhakiella capsodis (-)<br>strain N6PO6	na	mirid bug, *Capsodes infuscatus*	466	SAMN05216516_101630	516	SAMN05216516_101631	318	SAMN05216516_101632
Lactobacillus reuteri (+)<br>strain 480_44	na	*Mus musculus*	468	BBP10_01560	510<br>517	BBP10_01565<br>BBP10_01575	306<br>306	BBP10_01570<br>BBP10_04665#

							304	BBP10_07275#
Mesorhizobium soli (-)<br>strain JCM 19897	na	Forestal soil	464	C7I85_04950	517	C7I85_04945	314<br>308	C7I85_04940<br>C7I85_02950#
Morganella morganii (-)<br>subsp. morganii KT	NC_020418.1	Human	460<br>569	MU9_1665<br>MU9_1355 #	493	MU9_1664	309<br>335<br>308	MU9_1663<br>MU9_0411 #<br>MU9_2592 #
Obesumbacterium proteus (-)<br>strain DSM2777	NZ_CP014608.1	Faeces of wild boar	466	DSM2777_06325	515	DSM2777_06320	312<br>308<br>346	DSM2777_06315<br>DSM2777_00240#<br>DSM2777_13840#
Odoribacter splanchnicus (-)<br>DSM 20712	NC_015160	Human, abdominal abscess	465	Odosp_1307	538	Odosp_0380	321<br>304	Odosp_0379<br>Odosp_1505#
Oxalobacter formigenes (-)<br>*OXCC13* strain OXCC13	na	Human	465	BRW84_09560	524	BRW84_09570	316	BRW84_09565
Parabacteroides merdae (-)<br>ATCC 43184	NZ_AAXE00000000.2	Human	479	PARMER_03646	526	PARMER_03642	321<br>837<br>951	PARMER_03645<br>PARMER_02560#<br>PARMER_04124#
Paraburkholderia xenovorans (-)<br>LB400 [(8)]	NC_007951,<br>NC_007952,<br>NC_007953	Contaminated soil	461<br>461	Bxe_A3826<br>Bxe_C0551	506<br>506	Bxe_A3825<br>Bxe_C0552	304	Bxe_B1127
Photobacterium angustum (-)<br>LC1-200	na	seawater	466<br>555	BTO08_10405<br>BTO08_04580#	505<br>480	BTO08_10395<br>BTO08_18625 #	314<br>306	BTO08_10400<br>BTO08_11035#

Photobacterium damselae (-)<br>subsp. damselae strain KC-Na-1	NZ_CP021151.1	skin lesions on damselfish	466<br>549	CAY62_11315<br>CAY62_05970#	508	CAY62_11305	319<br>306	CAY62_11310<br>CAY62_11935#
Proteus mirabilis (-)<br>HI4320	NC_010554.1	Human	463	PMI1407	517	PMI1407	308	PMI0329
Providencia alcalifaciens (-)<br>Dmel2 [(9)]	NZ_AKKM00000000.1	Fruit fly, *Drosophila melanogaster*	466<br>391	OO9_16601<br>OO9_18596	512<br>518	OO9_16606<br>OO9_18591	311<br>308	OO9_18586<br>OO9_12295 #
Pseudomonas psychrophila (-) strain BS3667 [(10)]	NZ_LT629795.1	Petroleum sludge	465	SAMN04490201_1375	525	SAMN04490201_1373	314	SAMN04490201_1374
Serratia fonticola (-)<br>strain DMS 4576	NZ_CP011254.1	Human	466<br>466	WN53_13795<br>WN53_24805	512<br>512	WN53_13800<br>WN53_24810	307	WN53_03050
Shewanella halifaxensis (-)<br>HAW-EB4	NC_010334.1	Sediment	464<br>548	Shal_3043<br>Shal_2801 #	504<br>489	Shal_2708<br>Shal_2952 #	311<br>304	Shal_2709<br>Shal_1157 #
Shigella flexneri (-)<br>2a str. 301 [(11)]	NC_004337.2	Human	486<br>466<br>466	SF310_0690<br>SF301_3206<br>SF301_1869 #	486<br>511	SF301_0691<br>SF301_3205	310<br>308	SF301_2702<br>SF301_3386 #
Tannerella (-)<br>sp. 6_1_58FAA_CT1	na	Human	480	HMPREF1033_20619	509	HMPREF1033_20622	321	HMPREF1033_20620
Wohlfahrtiimonas chitiniclastica (-)<br>SH04	na	Fly, *Chrysomya megacephala*	458	F387_10770	489<br>479	F387_00701<br>F387_01771	307	F387_00700
Yersinia enterocolitica (-)<br>8081 [(12)]	NC_008800.1	Human	466	YE3693	518	YE3692	313	YE3691

							335<br>314	YE2500 #<br>YE3440 #
Yersinia ruckeri (-)<br>29473	na	Fish	467	DJ39_1792	531	DJ39_1793	312<br>308	DJ39_1794<br>DJ39_3036#

[a] The species and strains reported in this list are the most representative. The number near each species links to the list (provided below) of all the genomes where the same gene arrangement has been found. They are: [(1)] *A. indistinctus* 17126*; A. putredinis* DSM 17216*; A. finegoldii* DMS242 (but missing *gadB*) – [(2)] *B. oleiciplenus* YIT 12058; *B. massiliensis* B84634; *B. vulgatus*; *B. dorei* [(2a)]*B. caccae* ATCC 43185; *B. stercoris* ATCC 43183; *B. cellulosilyticus* WH2; *B. intestinalis*; *B. fluxus* – [(3)] *B. inopinata* BO1; Brucella *sp. Br2* 09RB8910; *B. ceti* L2/15; *B. pinnipedialis* BCCN06-44 – [(4)] *E. ictaluri* 93-146 – [(5)] *E. norvegicus.* – [(6)] *E. coli* 12264 (O76:H^-); *E. coli* 50588 (O8:H^-); *E. coli* DEC14D; *E. coli* E101; *E. coli* M718; *E. coli* STEC_94C – [(7)] *Fusobacterium nucleatum* subsp. polymorphum strain ChDC F30; *Fusobacterium periodonticum* strain KCOM 1263 – [(8)] the locus tags are of genes located on different chromosome, as indicated by the lettering A, B and C preceding each number – [(9)] *Providencia burhodogranariea* DSM 19968 possesses *gadBC* and *glsA* but far apart – [(10)] *P. fragi* P121– [(11)] *S. boydii; S. boydii* Sb227*; S. dysenteriae* 1617*; S. dysenteriae* 225-75*; S. dysenteriae* CD_74_112 – [(12)] *Y. frederiksenii* ATCC 33641; *Y. intermedia* ATCC 29909; *Y. kristensenii* ATCC 33638; *Y. kristensenii* ATCC 43969.

[b] In light grey are shown the species for which the genome sequence is not complete. For this reason in Fig. 5 and Fig. S6 the position of the genes in the genome (K, in kilobases) is not shown.

[c] (-) Gram –negative bacterium; (+) Gram –positive bacterium

[d] Refseq code: na = not available

[#] gene coding for an isoform of the relevant gene, but not reported in Figure 5 and Figure S6 because either not in close proximity of the structural genes coding for GadB, GadC, YbaS or YbaT (depending on the gene under consideration) or encoding a glutaminase enzyme that is likely or known to function at pH close-to-neutral.
