# Supplementary material for: The Glutaminase-Dependent Acid Resistance System: Qualitative and Quantitative Assays and Analysis of Its Distribution in Enteric Bacteria
Source: Front Microbiol. 2018 Nov 15;9:2869. doi: 10.3389/fmicb.2018.02869 (PMC6250119; doi:10.3389/fmicb.2018.02869)
Supplement: Supplementary file 3 [file Image_1.pdf]

Supplementary Material

The glutaminase-dependent acid resistance system: qualitative and quantitative assays and analysis of its distribution in enteric bacteria

Eugenia Pennacchietti [1], Chiara D'Alonzo[1], Luca Freddi [2], Alessandra Occhialini[2], Daniela De Biase[1*]

*** Correspondence:** Daniela De Biase: daniela.debiase@uniroma1.it

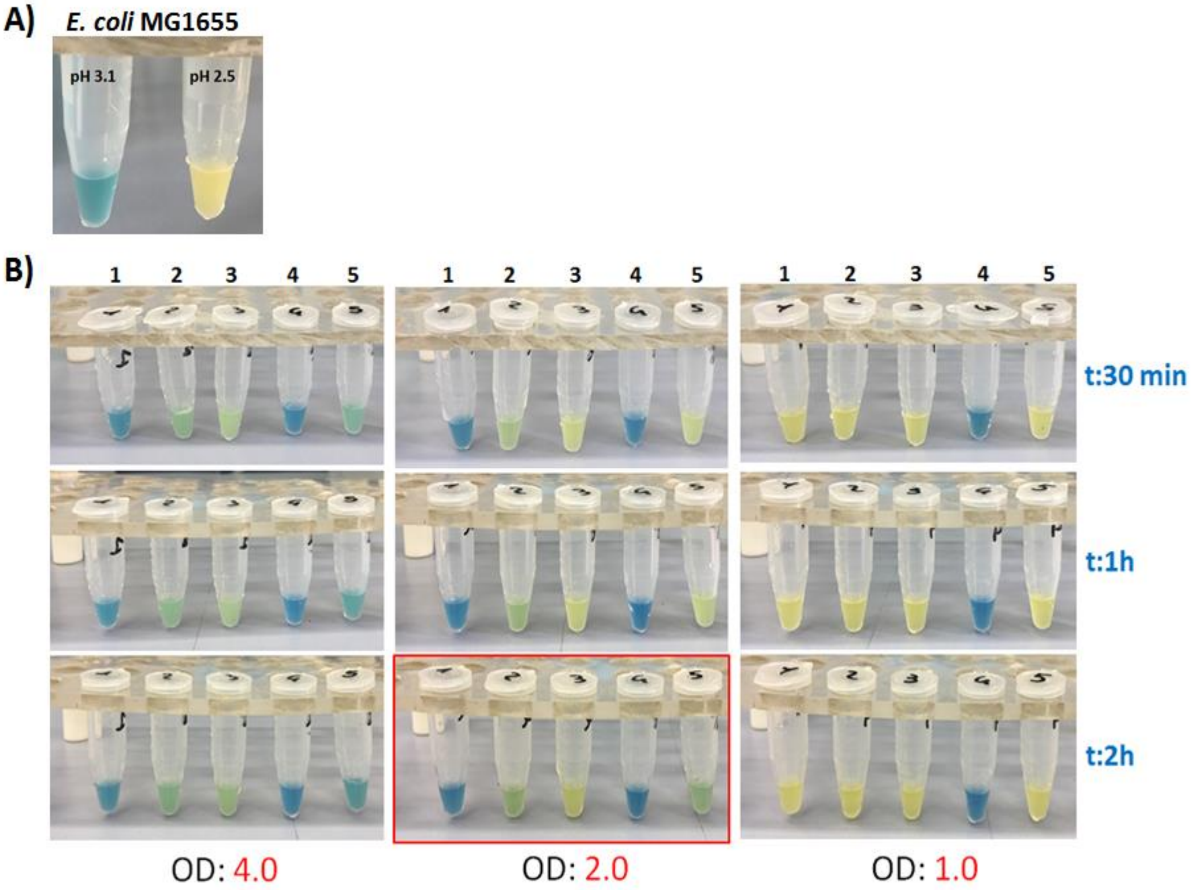

Figure S1. The GlsAssay in *Escherichia coli*. **A)** Preliminary control to find the best pH for the GlsAssay solution. The GlsAssay was performed on *E. coli* K12 MG1655 wild type strain at pH 3.1 (positive) and 2.5 (negative). The pH 2.5 is too extreme to perform the GlsAssay. In contrast, pH 2.8 is still acceptable (data not shown and data in Fig. 6B, right panel). **B)** The GlsAssay was performed with 500 µl of cells washed in physiological saline solution and brought to the indicated OD_{600} (horizontal panels), centrifuged again to separate from the physiological solution and resuspended in 150 µl of GlsAssay solution at pH 3.1. The assay was carried out for 30 min, 1 hour and 2 hours (vertical panels). The strains tested were 1) MG1655 wild type/pBBR; 2) MG1655Δ*gadA*-Δ*gadB*/pBBR; 3) MG1655Δ*ybaS*/pBBR; 4) MG1655Δ*ybaS*/pBBR-*ybaS_Ec*; 5) MG1655Δ*ybaS*/pBBR-*glsA_Bm*.
