# Supplementary material for: The Glutaminase-Dependent Acid Resistance System: Qualitative and Quantitative Assays and Analysis of Its Distribution in Enteric Bacteria
Source: Front Microbiol. 2018 Nov 15;9:2869. doi: 10.3389/fmicb.2018.02869 (PMC6250119; doi:10.3389/fmicb.2018.02869)
Supplement: Supplementary file 4 [file Image_2.pdf]

Supplementary Material

The glutaminase-dependent acid resistance system: qualitative and quantitative assays and analysis of its distribution in enteric bacteria

Eugenia Pennacchietti [1], Chiara D'Alonzo[1], Luca Freddi [2], Alessandra Occhialini[2], Daniela De Biase[1*]

*** Correspondence:** Daniela De Biase: daniela.debiase@uniroma1.it

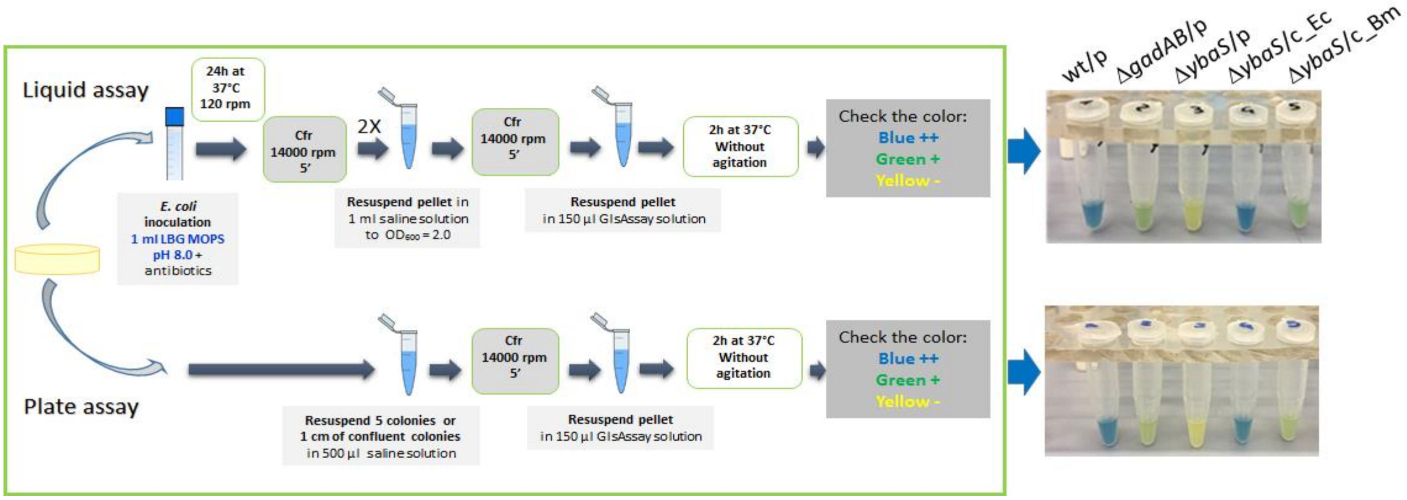

Figure S2. The GlsAssay "at glance". A schematic representation of the steps of the GlsAssay from liquid cultures and from colonies from plate. The pictures on the right show that similar results can be obtained with both approaches. In both cases the incubation was 2 hours at 37°C. The strain tested were: MG1655 wt/pBBR (wt/p); MG1655Δ*gadA*-Δ*gadB*/pBBR (Δ*gadAB*/p); MG1655Δ*ybaS*/pBBR (Δ*ybaS*/p); MG1655Δ*ybaS*/pBBR-*ybaS_Ec* (Δ*ybaS*/c_Ec); MG1655Δ*ybaS*/pBBR-*glsA_Bm* (Δ*ybaS*/c_Bm).
