# Supplementary material for: The Glutaminase-Dependent Acid Resistance System: Qualitative and Quantitative Assays and Analysis of Its Distribution in Enteric Bacteria
Source: Front Microbiol. 2018 Nov 15;9:2869. doi: 10.3389/fmicb.2018.02869 (PMC6250119; doi:10.3389/fmicb.2018.02869)
Supplement: Supplementary file 5 [file Image_3.PDF]

Supplementary Material

The glutaminase-dependent acid resistance system: qualitative and quantitative assays and analysis of its distribution in enteric bacteria

Eugenia Pennacchietti [1], Chiara D'Alonzo[1], Luca Freddi [2], Alessandra Occhialini[2], Daniela De Biase[1*]

*** Correspondence:** Daniela De Biase: daniela.debiase@uniroma1.it

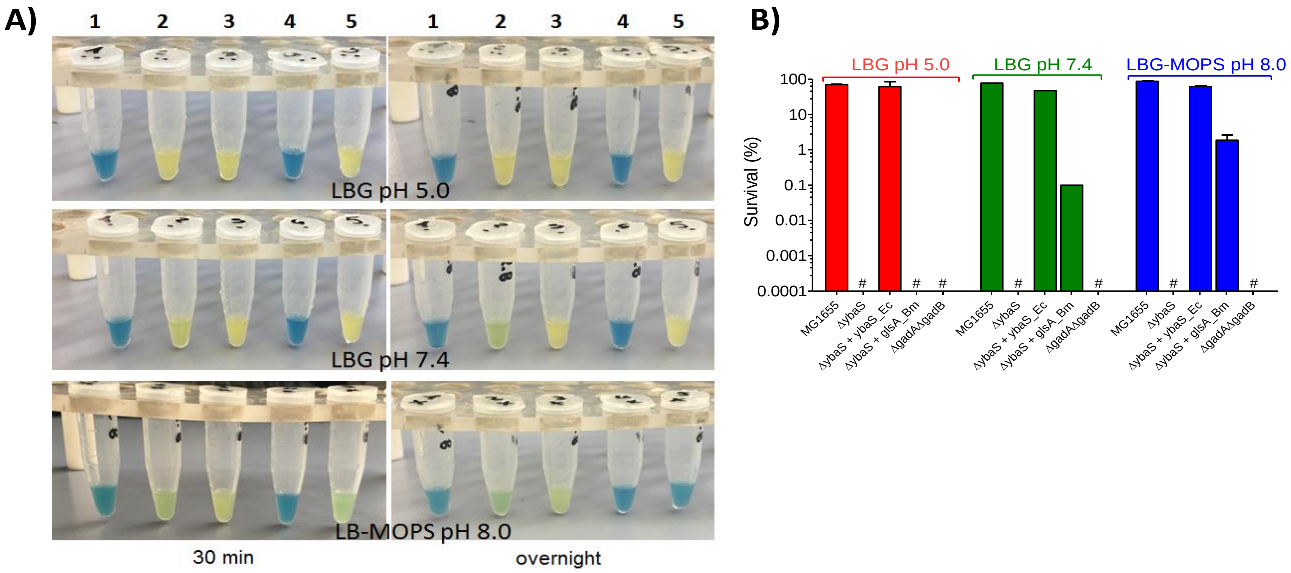

Figure S3. **Effect of the pH and composition of the growth medium on the expression of the acid-glutaminase, YbaS/GlsA. A)** The GlsAssay was used as a mean to find the best growth condition to induce the expression (and detect the activity) of the acid-glutaminase of *E. coli* and *B. microti*. The strains tested were: 1) MG1655 wild type/pBBR; 2) MG1655Δ*gadA*-Δ*gadB*/pBBR; 3) MG1655Δ*ybaS*/pBBR; 4) MG1655Δ*ybaS*/pBBR-*ybaS_Ec*; 5) MG1655Δ*ybaS*/pBBR-*glsA_Bm*. Prior to performing the assay, the bacteria were cultivated for 24 hours in the medium indicated. The GlsAssay was carried out for 2 hours before assessing the result. **B)** The same cultures were also assayed for AR in the presence of 3 mM Gln in EG pH 2.2 and incubated (statically) for 2 hours at 37°C. Residual viability following the acid challenge is expressed as % CFU/ml on a $\log_{10}$ scale, compared to bacteria present at time zero. The data represent the mean (SD) of 2-3 independent experiments. The hashtag indicates no survivors detected.
