# Supplementary material for: The Glutaminase-Dependent Acid Resistance System: Qualitative and Quantitative Assays and Analysis of Its Distribution in Enteric Bacteria
Source: Front Microbiol. 2018 Nov 15;9:2869. doi: 10.3389/fmicb.2018.02869 (PMC6250119; doi:10.3389/fmicb.2018.02869)
Supplement: Supplementary file 6 [file Image_4.PDF]

*Supplementary Material*

# The glutaminase-dependent acid resistance system: qualitative and quantitative assays and analysis of its distribution in enteric bacteria

**Eugenia Pennacchietti [1], Chiara D'Alonzo[1], Luca Freddi [2], Alessandra Occhialini[2], Daniela De Biase[1*]**

*** Correspondence:** Daniela De Biase: daniela.debiase@uniroma1.it

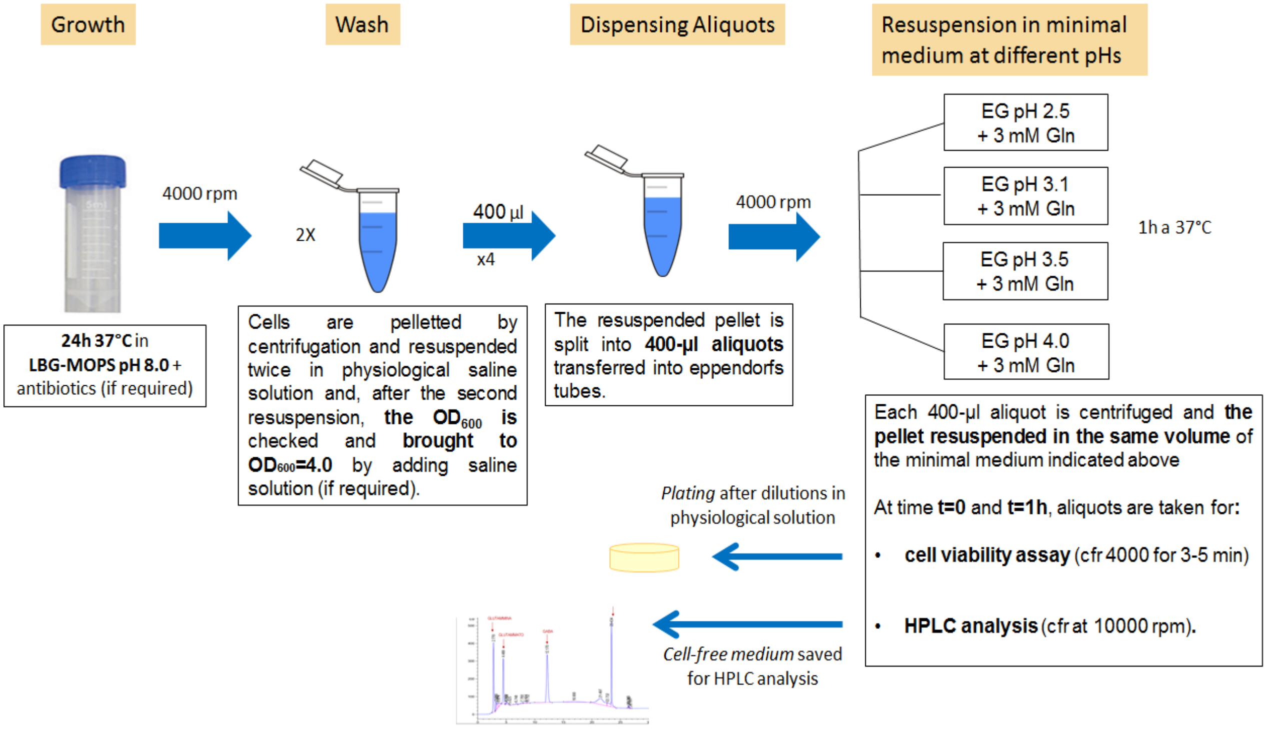


**Figure S4. Schematic representation of the steps of the quantitative HPLC assay.**
