# Supplementary material for: The Glutaminase-Dependent Acid Resistance System: Qualitative and Quantitative Assays and Analysis of Its Distribution in Enteric Bacteria
Source: Front Microbiol. 2018 Nov 15;9:2869. doi: 10.3389/fmicb.2018.02869 (PMC6250119; doi:10.3389/fmicb.2018.02869)
Supplement: Supplementary file 7 [file Image_5.PDF]

Supplementary Material

The glutaminase-dependent acid resistance system: qualitative and quantitative assays and analysis of its distribution in enteric bacteria

Eugenia Pennacchietti [1], Chiara D'Alonzo[1], Luca Freddi [2], Alessandra Occhialini[2], Daniela De Biase[1*]

*** Correspondence:** Daniela De Biase: daniela.debiase@uniroma1.it

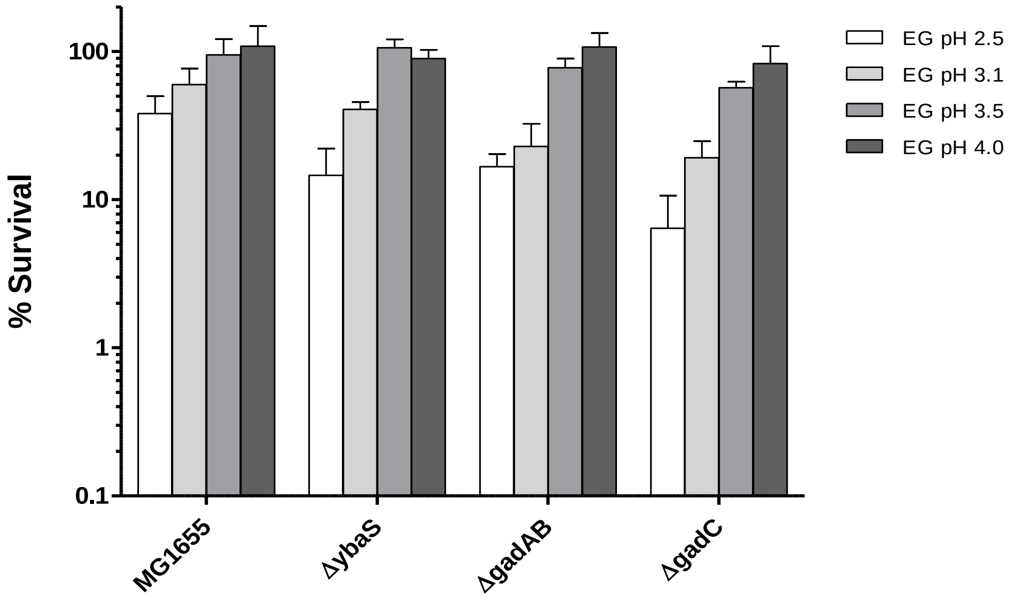

Figure S5. Percentage of survival following the exposure to EG minimal medium with glutamine at different pHs. The *E. coli* MG1655 wild-type strain and its Δ*ybaS*, Δ*gadA*-Δ*gadB* and Δ*gadC* isogenic mutant derivatives were tested for viability before and after the incubation for 1 hour in EG medium containing 3 mM Gln, at the indicated pH. Sequential dilutions in physiological solution were applied to determine the viability in the same samples that were used for the HPLC experiment reported in Figure 4. Data points represent means (SD) from 3 independent experiments.
