# Supplementary material for: The Glutaminase-Dependent Acid Resistance System: Qualitative and Quantitative Assays and Analysis of Its Distribution in Enteric Bacteria
Source: Front Microbiol. 2018 Nov 15;9:2869. doi: 10.3389/fmicb.2018.02869 (PMC6250119; doi:10.3389/fmicb.2018.02869)
Supplement: Supplementary file 8 [file Image_6.pdf]

Supplementary Material

The glutaminase-dependent acid resistance system: qualitative and quantitative assays and analysis of its distribution in enteric bacteria

Eugenia Pennacchietti [1], Chiara D'Alonzo[1], Luca Freddi [2], Alessandra Occhialini[2], Daniela De Biase[1*]

*** Correspondence:** Daniela De Biase: daniela.debiase@uniroma1.it

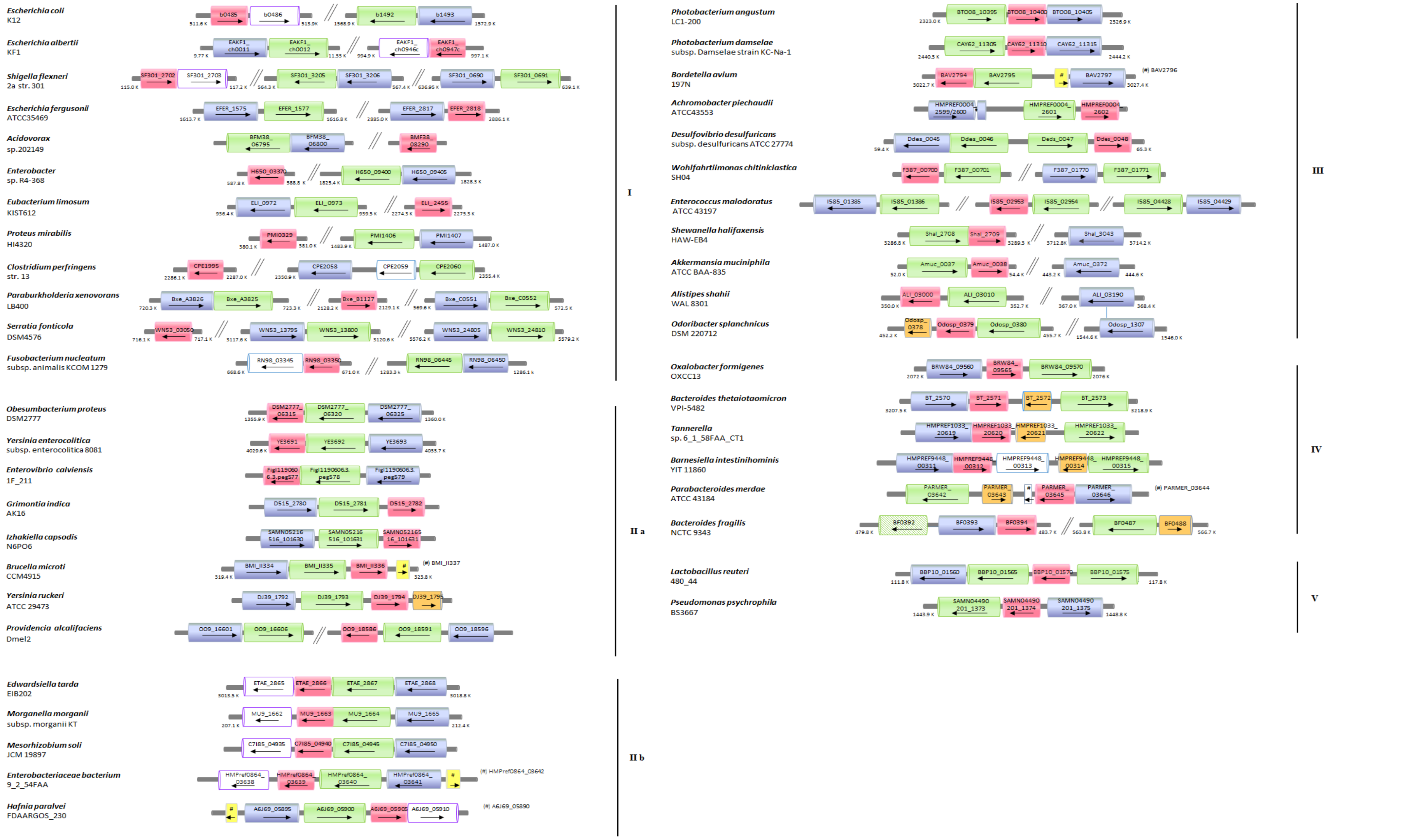

Figure S6. Schematic representation of the distribution of the genes coding for *ybaS/glsA*, *gadB* and *gadC* in different bacterial genomes. The bacterial species and strains where these genes were all found are reported in full. The arrow lengths and the relatives distance are proportional to the gene lengths and distances between adjacent genes, respectively. The corresponding locus tags are shown within each arrow. The homologous genes are represented in different colors: *ybaS/glsA*, in magenta; *gadC*, in green; *gadB*, in blue; *ybaT*, in white with

violet contour; *hdeA/B* (periplasmic chaperone) in yellow; putative potassium channel, in orange. The genes with putative functions are dashed in the same color as that of the genes with an assigned function. Other genes, possibly linked to AR, are in white with a cyan contour.
